# A generalised module for the selective extracellular accumulation of recombinant proteins

**DOI:** 10.1186/1475-2859-11-69

**Published:** 2012-05-28

**Authors:** Yanina R Sevastsyanovich, Denisse L Leyton, Timothy J Wells, Catherine A Wardius, Karina Tveen-Jensen, Faye C Morris, Timothy J Knowles, Adam F Cunningham, Jeffrey A Cole, Ian R Henderson

**Affiliations:** 1 School of Immunity and Infection, University of Birmingham, Edgbaston, Birmingham, B15 2TT, United Kingdom; 2 School of Cancer Sciences, University of Birmingham, Edgbaston, Birmingham, B15 2TT, United Kingdom; 3 School of Biosciences, University of Birmingham, Edgbaston, Birmingham, B15 2TT, United Kingdom

**Keywords:** Autotransporter, *Escherhichia coli*, Recombinant protein production, Secretion

## Abstract

**Background:**

It is widely believed that laboratory strains of *Escherichia coli*, including those used for industrial production of proteins, do not secrete proteins to the extracellular milieu.

**Results:**

Here, we report the development of a generalised module, based on an *E. coli* autotransporter secretion system, for the production of extracellular recombinant proteins. We demonstrate that a wide variety of structurally diverse proteins can be secreted as soluble proteins when linked to the autotransporter module. Yields were comparable to those achieved with other bacterial secretion systems.

**Conclusions:**

The advantage of this module is that it relies on a relatively simple and easily manipulated secretion system, exhibits no apparent limitation to the size of the secreted protein and can deliver proteins to the extracellular environment at levels of purity and yields sufficient for many biotechnological applications.

## Background

*Escherichia coli* is the preferred host for recombinant protein production (RPP) in both a research and industrial setting. The popularity of *E. coli* stems from attributes that include high growth rates in inexpensive media, high product yields, simple process scale-up and safety [1]. The choice of alternative hosts for RPP is predicated on the inability of *E. coli* to achieve adequate production of a target protein. A predominant reason for the selection of an alternative host is the apparent inability of laboratory strains of *E. coli* to secrete proteins to the extracellular milieu. Targeting recombinant proteins to the culture medium has several advantages over intracellular accumulation of the desired protein including overcoming problems with product toxicity, degradation, aggregation and incorrect folding [1,2]. In principle, it will reduce the number of downstream processing steps due to the ease of product recovery, the reduction in the number and quantity of process impurities and absence of laborious refolding experiments to isolate an active molecule [1].

Several non-specific strategies for extracellular accumulation of recombinant proteins have been developed for *E. coli* including genetically or chemically altering strains to promote protein leakage from the periplasmic space to the culture medium [3,4]. Unfortunately, this results in large numbers of process impurities in the form of lipids, polysaccharides and proteins derived from the periplasm space and outer membrane (OM). Conversely, if bacterial secretion systems could be manipulated to selectively secrete a desired target protein into the culture medium, in a controlled and predictable manner, it would drastically reduce costs and increase efficiency in bioprocessing [5]. The bacterial type 1, 2, 3 and chaperone-usher systems have been manipulated to secrete foreign proteins from *E. coli* and other Gram-negative bacteria [6-9]. However, their use for RPP is hampered by the debatable nature of the secretion signals, their molecular complexity (which results in species and/or substrate specificity) and the limited accumulation of the target protein [2]. Extensive genetic manipulation is required to make these systems tractable.

In contrast, the Type 5, or Autotransporter (AT), system has been utilised widely to successfully secrete a variety of heterologous target molecules to the bacterial cell surface in a process called Autodisplay [10-14]. ATs are widely distributed among Gram-negative bacteria [15-17]. The precursor protein contains an N-terminal signal sequence, which mediates Sec-dependent protein export into the periplasm, a passenger domain encoding the effector function and a C-terminal domain mediating translocation of the passenger domain across the OM [16,18,19]. The effector portion of the molecule displays functional and structural heterogeneity and can be substituted with heterologous proteins [14,16]. Whilst successful in delivering a diverse variety of molecules to the cell surface, the AT system has not been successfully adapted for accumulation of heterologous proteins in the culture medium. The system can be engineered to release the heterologous passenger protein into the culture medium with the use of a protease [14], but the use of such proteases is undesirable for production technologies. Here we demonstrate that an AT module can be utilised not only for cell surface display but also for the accumulation of heterologous proteins in the culture medium without the addition of exogenous protease.

## Results

### Extracellular accumulation of heterologous proteins

Other groups have demonstrated the utility of ATs for Autodisplay of heterologous proteins on the bacterial cell surface [14]. In this case the passenger domain remains covalently attached to the β-barrel translocating subunit. Unlike the ATs used for Autodisplay, cleavage of the passenger domain of the serine protease ATs of the *Enterobacteriaceae* (SPATEs) from their cognate β-barrel is effected by nucleophilic attack of β-barrel residues on a single residue in the α-helix [20]. As such, no exogenous protease is required for liberation of the passenger domain from the β-barrel and in theory the passenger domain can be completely replaced with a target protein. Thus, we hypothesised that the SPATEs could be used to target heterologous proteins to the extracellular milieu rather than the cell surface. To test this hypothesis, initial experiments focused on the *E. coli* SPATE protein, Pet [21]. When compared to other members of the SPATE family Pet possesses high amino acid sequence identity and structural similarity: the passenger domain consists of a central β-helical stem decorated with several discursive subdomains and is connected to the characteristic β-barrel by a short α-helical peptide (Additional file 1: Figure S1). The gene encoding Pet was synthesised *de novo* (Additional file 2: Figure S2) and cloned into the pASK-IBA33plus or pET22b expression vectors to create plasmid templates onto which the genes encoding heterolgous proteins could be grafted for further experiments.

**Figure 1:**
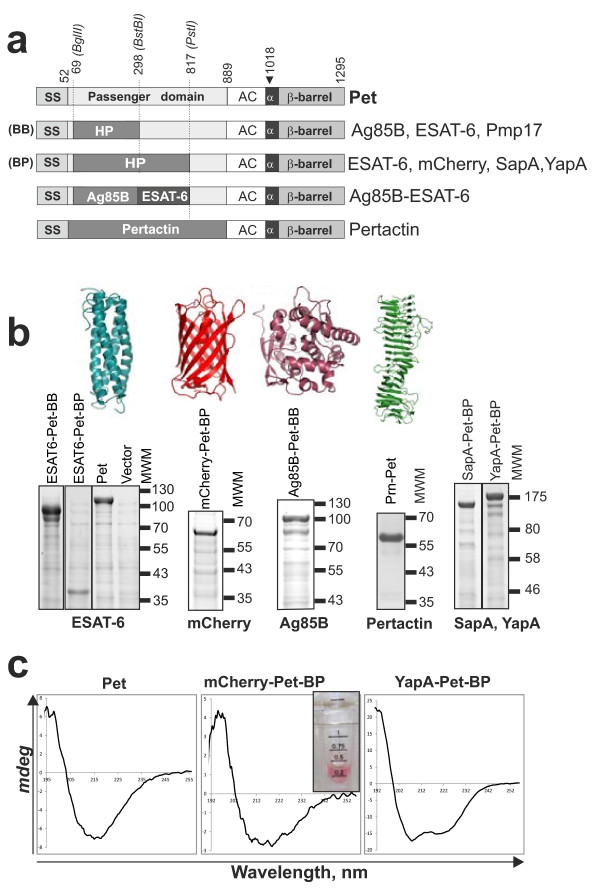
**AT-mediated accumulation of heterologous proteins in the culture medium. (A)** Schematic diagram of Pet fusion constructs. Heterologous protein insertions in the Pet passenger domain are shown by dark boxes marked HP or with the name of the protein, and are also listed on the right. Abbreviations BB and BP on the left refer to the type of protein fusion generated by insertion of foreign DNA into the *pet* gene between the restriction sites *Bgl*II and *BstB*I or *Bgl*II and *Pst*I, respectively. The co-ordinates above the figure are given for the amino acids derived from the *de novo* synthesised *pet* gene. The positions of these sites in the context of the quaternary structure are depicted in Additional file 1: Figure S1. The arrow at position 1018 denotes the cleavage site in the α-helix that effects release of the passenger domain into the culture medium. Modification of this site results in surface display of molecules (Figure 2). The abbreviations SS, AC and α denote the positions of the signal sequence, autochaperone domain and α-helix, respectively. **(B)** The presence of secreted heterologous proteins in the culture medium was detected by SDS-PAGE or immunoblotting with anti-Pet. Equivalent volumes of medium were analysed. The structures of several heterologous proteins are depicted (not to scale). **(C)** Investigation of the folded state of secreted heterologous fusion proteins. Far-UV CD spectra of Pet and several heterologous proteins are shown in millidegrees (*mdeg*). mCherry-Pet-BP harvested from the culture supernatant is shown.

To test the ability of Pet to mediate secretion of heterologous proteins into the culture medium we chose proteins with distinctive size, structural and functional signatures. These included the secreted portions of (a) Pertactin from *Bordetella pertussis,* a component of the acellular whooping cough vaccine (43.9 kDa), (b) YapA, a surface protein from *Yersinia pestis* (105.2 kDa)*,* (c) Pmp17, a polymorphic surface protein from *Chlamydophila abortus* (40 kDa), (d) SapA, a putative surface protein from *Salmonella enterica* serovar Typhimurium (60.7 kDa), (e) the red fluorescent protein mCherry, a derivative of *Discosoma* sp DsRed (26.7 kDa), (f) the predicted secreted esterase Ag85B , a putative *Mycobacterium tuberculosis* vaccine candidate (34.6 kDa) and (g) ESAT-6 the major diagnostic marker from *M. tuberculosis* (10 kDa) [17,22-25]. DNA encoding the heterologous proteins was synthesized *de novo* after codon optimization for expression in *E. coli* (Additional file 2: Figure S2). Previous Autodisplay experiments suggested the N-terminal portion of AT passenger domains were not required for secretion of heterologous proteins to the bacterial cell surface [14]. To verify if this was also the case for proteins released into the culture medium, different parts of the *pet* gene were replaced in-frame with heterologous DNA to give rise to fusion proteins as shown in Figure 1A. All of the chimeric proteins were secreted into the culture medium as soluble proteins at levels stoichiometrically similar to the wild-type Pet protein (Figure 1B) confirming the N-terminal portions of the passenger domain are not required for secretion. Similarly, the cleaved β-barrel accumulated in the OM at levels indistinguishable from wild-type protein (Additional file 3: Figure S3). To authenticate the identities of all secreted proteins, bands were excised from polyacrylamide gels and subjected to mass spectrometry; the appropriate protein was confirmed in each case (Additional file 4: Table S1). Finally, yields of Pertactin and ESAT-6 from Pet fusion constructs in shake flasks were calculated and accounted for as much as 5% total cell protein, depending on the expression strain and conditions. For ESAT6-Pet variants and Pertactin-Pet concentrations of 5.4 and 1 mg/l respectively were achieved after expression in *E. coli* BL21*.

### Heterologous proteins are secreted

**Figure 2:**
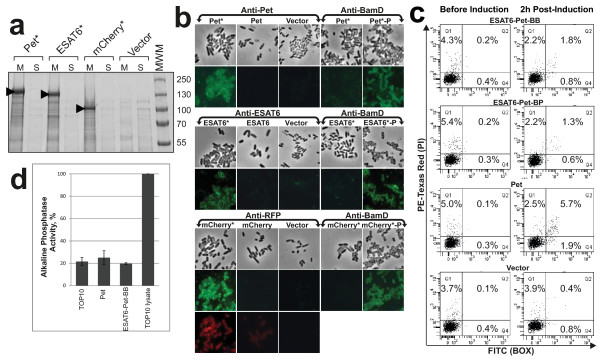
**Monitoring cellular integrity of the*E. coli*host strain expressing AT chimeras. (A)** SDS-PAGE analysis of OM (M) and culture supernatant (S) fractions derived from *E. coli* TOP10 expressing Pet*, mCherry*and ESAT6*. Non cleaved species are denoted by arrows. Molecular weight markers (MWM, kDa) are indicated to the right of the panel. OM fractions demonstrating the presence and absence of the β-domain are shown in Additional file 6: Figure S5. **(B)***E. coli* cells expressing empty vector, Pet*, mCherry*, ESAT6* and their cleaved parents were harvested 2 h after induction and subjected to indirect immunofluorescence using the indicated antibody. For each population expressing a cleavage deficient variant, a sample was divided in two: one half was probed with an antibody to the periplasmic protein BamD, while the other half was permeabilised (−P) and subsequently probed with the same antibody. Corresponding fields are also shown by phase contrast microscopy. For the mCherry constructs panels showing mCherry derived fluorescence are shown. **(C)** The integrity of *E. coli* host cells expressing wild type Pet and secreted ESAT6-Pet fusions were assessed by staining with BOX and PI prior to and 2 h post induction. Q3 represents the population that is viable and healthy and did not label with either stain. Q2 represents cells that stain with both stains and are no longer viable. Q4 (BOX-positive) represents cells with impaired membrane potential suggesting compromised membrane integrity. **(D)** Periplasmic leakage from *E. coli* TOP10 cultures secreting Pet or ESAT6-Pet fusion proteins was assessed by measuring (2 h after induction) the activity in the culture medium of the periplasmic enzyme alkaline phosphatase. Clarified whole cell lysate was used as a positive control and the wild-type plasmid-free strain as a negative control. There is no significant difference between the negative control and culture medium derived from strains expressing Pet or ESAT6-Pet-BB. In contrast, culture medium from both constructs displayed activity significantly less than the positive control. Absorbance measurements are derived from equal volumes of culture and are normalised relative to positive control (in %). The error bars represent standard error for two independent data sets.

To demonstrate that the presence of heterologous proteins in the culture medium was due to secretion rather than leakage from the periplasm or cell lysis, we examined the cellular location of mCherry and ESAT-6. Recently, we described a mutation in Pet (Pet*) that disrupts the interdomain cleavage site such that the passenger domain is completely translocated to the cell surface but remains covalently attached to the β-domain [26]. This mutation was introduced into mCherry-Pet-BP and ESAT6-Pet-BB to create mCherry* and ESAT6*, respectively. In each case no passenger domain accumulates in the culture medium and full length versions can be detected in the OM (Figure 2A and Additional file 5: Figure S4). Immunofluorescence studies of bacteria expressing Pet*, mCherry* and ESAT6* with specific antibodies revealed surface localisation of passenger domains whereas with the native cleavage site there was negligible staining and protein accumulated in the medium (Figure 2B and Figure 1B). Flow cytometry confirmed these observations (Additional file 5: Figure S4). These experiments demonstrate that the heterologous fusions are expressed and actively translocated to the cell surface before cleavage. Crucially, probing with antibodies directed at the periplasmic protein BamD [27] revealed labelling was not due to ingress of antibodies into the bacterial cell since cells did not label unless permeabilised by chemical treatment (Figure 2B); hence secretion occurred without major loss of membrane integrity. To ensure proteins were not released into the culture medium by cell lysis upon induction of expression, staining with propidium iodide (PI) and Bis-(1,3-dibutylbarbituric acid) trimethine oxonol (BOX) was used to assess cell viability and the integrity of the cell envelope of bacteria secreting heterologous fusions. Importantly, flow cytometry analyses of cultures expressing ESAT6-Pet-BB, ESAT6-Pet-BP and Pet proteins revealed that the majority of cells remain healthy and alive during protein secretion with only negligible increases in the number of BOX- or PI + BOX-positive cells after induction of protein expression compared to uninduced cultures (Figure 2C). Finally, assays for alkaline phosphatase, a periplasmic enzyme, demonstrate no leakage of periplasmic proteins after expression of heterologous fusions (Figure 2D). Taken together these data indicate that the presence of secreted proteins in the culture media is not due to cell lysis or periplasmic leakage, but active secretion. Additionally, the presence of ESAT-6 and fluorescent mCherry on the bacterial cell surface of cultures expressing mCherry* and ESAT6*, indicated the Pet AT-module, lacking the cleavage site, can also be used for autodisplay of functional proteins (Figure 2B).

### Secreted proteins are soluble, folded and can be modified

To be useful as a method of RPP, the AT system must be able to secrete soluble, folded and functional proteins into the culture medium. To test if the chimeric proteins were natively folded after secretion, we harvested the proteins from the culture supernatant fractions and subjected them to analysis by circular dichroism (CD) (Figure 1C). The structure of YapA is unknown, however bioinformatic analyses predicted YapA to possess a mixed α-helical/β-strand conformation whereas structural data reveal mCherry adopts a β-barrel conformation [24]. CD spectra of YapA showed minima at 222 nm and 208 nm and maxima at 195 nm indicative of a folded protein with mixed α-helical/β-strand content. Consistent with their natively folded β-strand conformations, CD spectra for Pet and mCherry show minima at 218 nm and maxima at 195 nm. Additionally, mCherry purified from the culture supernatant fraction shows fluorescence indicating a folded protein with functional activity (Figure 1C).

**Figure 3:**
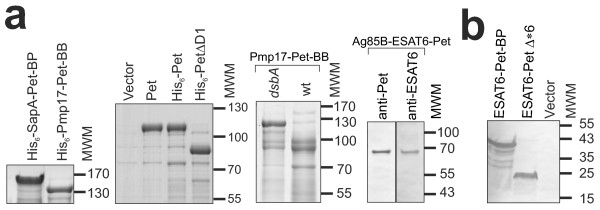
**Modification of the Pet-AT secretion platform. (A)** SapA, Pmp17, Pet and a Pet derivative (PetΔD1) lacking the serine protease domain were modified to add a His_6_-Tag to the N-terminus of the secreted passenger domain. The His-tagged Pmp17 protein was expressed in an *E. coli* TOP10 *dsbA* strain and the rest of proteins were produced in the wild-type *E. coli* TOP10. In all cases the proteins are well secreted. The cysteine-containing Pmp17 was expressed in *E. coli* TOP10 (wt) and a *dsbA*^*-*^ derivative. A full length protein is present in the *E. coli* TOP10 *dsbA*^-^ derivative but not the *E. coli* TOP10 parent strain. Break down products are apparent and correspond to proteins with a truncated N-terminus. A multicomponent construct was created by fusing DNA encoding Ag85 to ESAT-6 and Pet (see Figure 1A) to encode a single polypeptide chain contiguous with the AT-translocation unit. This latter chimera was detected in the culture supernatant with antibodies directed at Pet and ESAT-6. Equivalent amounts of culture supernatant fractions were analysed by SDS-PAGE. **(B)** Secretion of heterologous fusions from *S.* Typhimurium. Culture medium from *S. enterica* SL1344 strains expressing ESAT6-Pet-BP and ESAT6-PetΔ*6 (see Figure 4) were harvested and analysed by SDS-PAGE and detected by immunoblotting with a polyclonal antibody to ESAT-6. In all SDS-PAGE gels the positions of the molecular weight markers (MWM, kDa) are depicted at the right side of the panel. The equivalent OM fractions demonstrating the presence of the cleaved β-barrel are shown in Additional file 5: Figure S4.

Having established the Pet AT system can support the specific secretion of some heterologous proteins in a folded and functional state, we investigated whether the system could secrete modified chimeras consisting of proteins with disulphide bonded cysteine residues, purification tags or multiprotein complexes. Proteins targeted for secretion by the AT mechanism have to traverse the periplasm, a highly oxidising cellular compartment where formation of disulphide bonds between cysteine residues is catalysed by DsbA [28]. Pmp17-Pet-BB, possessing 7 cysteines, was expressed in *E. coli* TOP10 *dsbA* and accumulated in the culture medium at levels consistent with wild-type Pet (Figure 3A). No full-length protein could be detected in wild-type *E. coli* TOP10. However, degradation products were observed in the extracellular milieu. These products are consistent with a degraded passenger domain lacking the ca. 24 kDa N-terminal region containing the cysteines and based on previous experiments is the result of the action of the periplasmic protease DegP [26,29]. When the N-terminal region containing the cysteines is removed by DegP-mediated degradation the disulphide bonds cannot form and secretion is no longer stalled. These results are consistent with observations made for Pet and other ATs that demonstrate disulphide bond formation and partial folding of native or heterologous polypeptides hinders their secretion [26,29,30]. Fusion tags might be desirable for downstream purification applications. N-terminal addition of a His_6_-tag to the SapA and Pmp17 passenger domains, as well as on wild-type and truncated Pet derivatives, did not interfere with secretion of target proteins to the extracellular milieu (Figure 3A). Previously, we demonstrated the system is capable of secreting proteins with HA- and FLAG-tags [26]. Notably, the multicomponent chimera containing both Ag85B and ESAT-6 proteins could also be secreted by Pet (Figure 3A). To determine whether the AT module can be utilised for production of heterologous proteins in other Gram-negative bacteria we investigated expression of ESAT6-Pet-BP and ESAT6-PetΔ*6 (see below) in *S.* Typhimurium SL1344 and its avirulent derivative SL3261. In both cases, ESAT-6 was secreted into the culture medium at levels similar to that for Pet (Figure 3B). These data demonstrate the versatility of the Pet AT-module for secreting proteins and multiprotein complexes into the culture medium.

### Determination of the minimal construct mediating secretion

**Figure 4:**
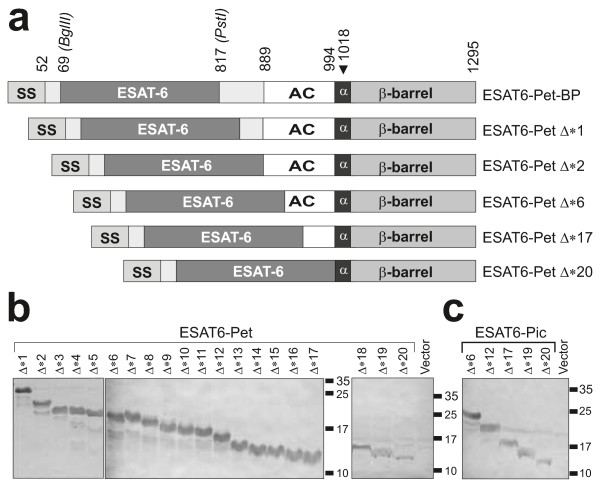
**Identification of the minimal AT module permitting secretion of heterologous proteins to the culture supernatant fraction. (A)** Schematic of ESAT6-Pet-BP protein fusion and some truncations created to determine the minimal C-terminal Pet fragment capable of ESAT-6 secretion. The Δ*1, Δ*2, Δ*6, Δ*17 and Δ*20 Pet truncations are shown while for simplicity the intermediate variants Δ*3–Δ*5, Δ*7–Δ*16, Δ*18 and Δ*19 are omitted. Abbreviations are the same as in Figure 1. **(B and C)** Detection by western immunoblotting of ESAT6-Pet (B) and ESAT6-Pic (C) chimeras expressed in *E. coli* TOP10. The TCA-precipitated culture supernatants were analysed by SDS-PAGE and probed with polyclonal anti-ESAT6. The equivalent OM fractions demonstrating the presence of the cleaved β-barrel in the OM are shown in Additional file 6: Figure S5.

The constructs described above possessed at least 100 amino acids upstream of the predicted Pet β-barrel. This stretch of amino acids encompasses the α-helical peptide spanning the β-barrel lumen and the Autochaperone (AC) domain. Previously, the AC domain has been implicated in secretion of passenger domains, where contemporaneous folding of the β-helix and a Brownian ratchet mechanism provide the vectorial impetus for secretion [31,32]. Here we sought to determine the precise length of the minimal functional translocation domain for Pet and determine whether the AC-domain is required for secretion of heterologous proteins to the growth medium. To this end, we examined secretion of ESAT-6-Pet-BP and 20 derivatives harbouring sequential truncations (Figure 4 and Additional file 6: Figure S5). All Pet derivatives, including the smallest variant tested that contains only the predicted α-helix and the downstream β-barrel domain, could sustain ESAT-6 secretion into the culture medium (Figure 4). ESAT-6 secreted by this Pet fragment contains only the 9 amino acids of the wild-type Pet passenger domain α-helix that are adjacent to the cleavage site. Two recent reports implicated a conserved tryptophan residue (W985) in the AC domain in secretion of some SPATEs [33,34]. Interestingly, ESAT6-PetΔ*17 to Δ*20 proteins lack the predicted Pet AC domain altogether but are well secreted (Figure 4). To further test a role for W985 in secretion, this amino acid and three other conserved and closely positioned residues (I983, L987 and G989) were mutated to Ala and Lys in the secretion-competent ESAT6-PetΔ*6. All mutated proteins were secreted into the growth medium as efficiently as the ESAT6-PetΔ*6 and retained a cleaved β-domain in the OM (Additional file 7: Figure S6). These data further support the view that the AC domain is not required for secretion *per se* but is essential for folding of native AT polypeptides.

Next we investigated whether other SPATE proteins could support secretion of heterologous proteins in a manner analogous to Pet. Pic [35] belongs to a clade of the SPATEs that is evolutionarily distinct from that harbouring Pet. Alignment of Pet and Pic protein sequences from the beginning of the predicted AC domains shows 68% identity and 80% similarity (Additional file 8: Figure S7). Based on the alignment we constructed ESAT6-Pic fusions containing C-terminal Pic fragments equivalent to those for ESAT6-Pet variants. All ESAT6-Pic fusions were efficiently secreted (Figure 4C) allowing us to conclude that β-barrels from SPATE proteins other than Pet can be used to secrete proteins to the culture medium.

## Discussion

*E. coli* was the first organism to be used for industrial scale production of recombinant proteins. Since then, a large number of proteins produced in *E. coli* have successfully reached the market including human interferons, interleukins, and granulocyte stimulating factor. However, the dominance of *E. coli* in industrial bioprocessing is waning with only ca. 40% of new recombinant protein pharmaceuticals being produced using prokaryotic cells. The diminution of *E. coli* is strongly related to that fact that strains of *E. coli* used for industrial scale protein production do not effectively secrete proteins into the extracellular environment. Solutions to this problem would effectively reposition *E. coli* as the host of choice for industrial recombinant protein production. To achieve selective accumulation of recombinant proteins in the extracellular milieu, a Gram-negative protein secretion system must be harnessed. While secretion of heterologous proteins through the Type 1–3 systems has been achieved, the complexity of these systems limits the nature and size of the proteins that can be targeted for secretion. The work described here demonstrates that an AT module, based on SPATE proteins, can be used for the targeted secretion of chimeric proteins into the culture media of Gram-negative bacteria. Importantly, we have demonstrated that this platform may be used for the specific accumulation of folded heterologous proteins with functional, structural and size heterogeneity and for multicomponent complexes (Figure 1). The ability to selectively accumulate target proteins in the culture medium, away from the majority of the process impurities associated with expression in other compartments, makes this system attractive for adoption in industrial RPP applications since extracellular expression of proteins in a folded active form enables simplification of the purification process and significantly reduces downstream processing. However, effective utilisation of the AT module for generalised RPP necessitates achieving yields of target proteins at industrial scale and at concentrations competitive with alternative technologies. Importantly, the yields achieved here are consistent with levels achieved for other *E. coli* protein secretion systems [1,36]. However, in the experiments conducted here, only ~ 50% of the expressed protein is targeted to the extracellular milieu, the remainder accumulating at the outer membrane. Furthermore, these yields were obtained in small-scale non-optimised conditions. Thus, an optimised secretion platform, in a controlled optimised fermentation process, would be expected to generate higher protein yields.

Live attenuated bacterial vectors offer the opportunity to deliver vaccine candidates inside human cells thereby eliciting a protective immune response against both infectious and non-infectious disease e.g. tumours. While the induction of antibody has been demonstrated in many animal models, the anticipated induction of cell-mediated immunity has been disappointing [37]. There are several reasons for this including (1) inefficient production of the recombinant heterologous antigen and (2) after invading professional phagocytic cells the live vector remains in the phagosome such that its antigens do not reach the cytosolic processing pathway. Surface display of AT-fusions in live-attenuated vaccine strains offers potential to overcome these problems [14]. Here we have demonstrated that the AT-module can be used in conjunction with live-attenuated Salmonella strains and demonstrated the successful surface display of folded functional heterologous proteins (Figure 2 and 3). Unfortunately, previous experiments using surface display have also failed to induce substantial protective immunity [14]. However, the ability of live attenuated vectors to secrete antigens intracellularly may enhance presentation of the antigens to the immune system and provide the desired protective response [38]. Thus, our demonstrated expression of secreted heterologous protein constructs in live attenuated Salmonella strains offers the possibility of developing a platform for the delivery of multivalent vaccines based on the continuous secretion of proteins *in vivo* in which significant cell mediated immunity is generated.

Finally, this work reveals novel insight into the biology of the AT secretion system. Several articles have described the importance of specific amino acid residues for the secretion of passenger domain proteins notably residues present in the AC domain [33,34]. Indeed, recent investigations of BrkA suggested that during secretion portions of the AC domain are sequestered by a specific domain within the β-barrel which initiates translocation of the passenger domain to the external environment [39]. Work provided here clearly demonstrates that the AC domain is not essential for secretion since the smallest secretion-competent constructs completely lack this domain and mutations within the domain do not affect secretion levels (Figure 4 and Additional file 8: Figure S7). These investigations reinforce the concept that the AC domain is required specifically for folding of the β-helical passenger domain, although folding of the passenger domain may enhance the rate at which secretion occurs [30].

## Conclusions

In conclusion, we have developed a versatile platform, based on an AT module, which can be used for secretion of heterologous fusion proteins to the culture medium in a soluble folded form. Additionally, this system can be used for surface display of heterologous fusions on the bacterial cell surface.

## Methods

### Bacterial strains and growth conditions

*E. coli* strains TOP10 (Invitrogen), NEB 5αF’I^q^ and JM110 (NEB) were used for cloning. *E. coli* TOP10, TOP10 *dsbA*[26] and BL21* (Novagen) and *S. enterica* SL1344 and SL3261 strains [40] were used for protein expression and secretion. Bacteria were grown at 37°C in Luria-Bertani liquid or solid media supplemented with carbenicillin (100 and 80 μg/ml, respectively) or kanamycin (50 μg/ml) when appropriate. Protein expression was induced by adding anhydrotetracycline (200 μg/l) or IPTG (0.5 mM) as appropriate and the cultures were incubated for a further 1.5–2 h.

### General molecular biology techniques and plasmid construction

Recombinant DNA manipulations were described elsewhere [41]. Phusion High-Fidelity DNA polymerase (Finnzymes), DNA modifying enzymes, plasmid mini-prep and PCR/gel extraction kits (Fermentas, Qiagen and NEB) were used according to the manufacturer’s instructions. Oligonucleotides were synthesized by Alta Bioscience and Eurogentec. Sequencing, mass spectrometry, flow cytometry and gel imaging/densitometry were done using the University of Birmingham Functional genomics facility. Codon optimization for the most commonly used codons and *de novo* synthesis of DNA was done by GenScript, GenArt or Epoch Life Science. Alignments were generated with ClustalX [42] and phylogenetic trees generated with Geneious software (http://www.geneious.com/).

Plasmids used in this study are listed in Additional file 9: Table S2. Primers used for PCR are listed in Additional file 10: Table S3. To construct pASK-Pet, the *pet* gene was PCR-amplified from pBAD-Pet with BsaI-pet-F and HindIII-pet-R primers and cloned between *Bsa*I*/Hind*III sites in pASK-IBA33plus (IBA BioTAGnology). pET-Pet was constructed by cloning the *pet* gene into the *Nde*I*-Hind*III sites of pET22b. pASK-His_6_-Pet and pASK-His_6_-Pet-ΔD1 were constructed by replacing the *Sac*I*-Bgl*II or *Sac*I*-BstB*I *pet* fragment in pASK-Pet with an amplicon generated by PCR with SacI-pet-F and PetSS-BglII-AflII-BstBI-R primers, the latter encoding a His_6_-tag sequence. To construct *pet* chimeras the heterologous genes were amplified by PCR using appropriate primer pairs and relevant DNA templates listed in Additional file 2: Figure S2. The PCR-amplified heterologous genes were cloned between *Bgl*II*/BstB*I and *Bgl*II*/Pst*I sites in the *pet* gene in pASK-Pet, pASK-Pet* or pET-Pet. pASK-Ag85B-ESAT6-Pet was constructed by inserting PCR-amplified *esxA* gene (ESAT-6) between *BstB*I*-Pst*I sites in pASK-Ag85B-Pet-BB. Constructs pASK-ESAT6-Pet Δ*1 to Δ*20 were made by replacing the *Pst*I*-Hind*III fragment in pASK-ESAT6-Pet-BP with the shorter *pet* gene fragments generated by PCR with one of the forward primers (PstI-TSYQ-del1-F to PstI-YKAF-del20-F) and HindIII-pet-R as a reverse primer. Equivalent constructs encoding ESAT6-Pic chimeras were generated by replacing the *Pst*I*-Hind*III *pet* fragment in pASK-ESAT6-Pet-BP with the *pic* fragment amplified from pPic1 [35] using one of the forward primers SbfI-FKAG-Pic-del6-F to SbfI-YKNF-Pic-del20-F) and HindIII-Pic-end-R as a reverse primer. Codons encoding I974, W985, L987 and G989 were mutated by site directed mutagenesis using primers BglII-ESAT6-F and HindIII-pet-R as previously described [43]. All constructs generated in this study were sequenced to confirm the veracity of the nucleotide modifications.

### Preparation and analysis of proteins

Proteins were visualised by Coomassie staining after SDS-PAGE on standard [44] or precast (Precise 4-20% Tris/HEPES, ThermoFisher; NuPAGE 4-12% Bis-Tris/MES, Invitrogen) polyacrylamide protein gels or by western immunoblotting as previously described [45]. Rabbit polyclonal antibodies against Pet passenger domain (1:5000 dilution) [46], ESAT-6 (Abcam; 1:2000 dilution) and mCherry (anti-RFP, Abcam; 1:2000) were used for western immunoblotting. Secondary alkaline phosphatase-conjugated goat anti-rabbit antibodies and NBT/BCIP (Nitro blue tetrazolium chloride/5-Bromo-4-chloro-3-indolylphosphate) substrate were purchased from Sigma. Protein concentrations were determined spectrophotometrically and by SDS-PAGE comparisons with known quantities of purified protein: Bovine serum albumin, Ovalbumin and Lysozyme (Sigma).

Cellular fractions were prepared essentially as described previously [44]. His_6_-tagged proteins were purified by affinity chromatography on Ni-agarose following manufacturer’s instructions (WebScientific). Briefly, 400 ml cultures were grown and protein expression was induced as described above. Culture supernatants were harvested and sterilised as above, supplemented with 1 mM PMSF and then concentrated through Vivaspin centrifugation device (Sartorius). The concentrated supernatant fractions were passed over a Ni-agarose affinity chromatography column under native conditions using 50 mM sodium phosphate, 500 mM NaCl, 10 mM imidazole (pH7.5) as binding buffer and 50 mM sodium phosphate, 500 mM NaCl, 500 mM imidazole (pH7.5) as elution buffer.

To test viability, bacterial cells (~10^5^–10^6^ cells/ml) were diluted in 1 ml filter-sterilised Dulbecco’s PBS supplemented with 10 μl of working solutions of PI and BOX (5 and 10 μg/ml respectively; Sigma) and analysed immediately on FACSAria II (BD Biosciences) using 488 nm laser [47]. Side and forward scatter data and fluorescence data from 10^4^ particles were collected. Optical filters used to measure green and red fluorescence were 502LP, 530/30BP (FITC) and 610LP, 616/23BP (PE-Texas Red), respectively. To analyse surface localisation of proteins by indirect flow cytometry, cells were washed in PBS and incubated at RT with 1% BSA in PBS. Cells were then incubated for 1 h at RT with primary antibody diluted in PBS (anti-Pet, 1:500; anti-ESAT6, 1:500; anti-mCherry, 1:800) followed by 3 PBS washes and final incubation with Alexa Fluor® 488 goat anti-rabbit IgG (1:500; Invitrogen). Cells were washed as before and analysed on a FACSAria II as above.

Proteins in live or fixed bacterial cells were detected by indirect Immunofluorescence as previously described [26]. Cells were visualized using either phase contrast or fluorescence using a Leica DMRE fluorescence microscope-DC200 digital camera system. Exposure time was 118 ms. The Garen and Levinthal [48] assay of Alkaline Phosphatase activity was used based on conversion of *p*-nitrophenylphosphate (pNPP) substrate into yellow product with absorbance at 410 nm. Far-UV CD measurements from 190 to 260 nm were collected on a JASCO J-715 spectropolarimeter at room temperature, as described previously [26]. Protein structures were modelled in Swiss-Model [49] or Phyre [50] and were visualised using PyMol (http:\\http://www.pymol.org). Secondary structures were predicted with PsiPred [51].

## Abbreviations

RPP, recombinant protein production; AT, Autotransporter; OM, Outer Membrane; SPATEs, Serine Protease Autotransporter of the Enterobacteriaceae; AC, Autochaperone; BOX, Bis-(1,3-dibutylbarbituric acid) trimethine oxonol; PI, Propidium Iodide.

## Competing interests

The authors declare that they have no competing interests. The work described in this article has been submitted for patent protection by the University of Birmingham.

## Author contributions

IRH, JAC and AFC designed the project. DLL, YRS and KT designed and constructed the expression vectors. YRS, DLL and TJK purified proteins and performed CD analyses. YRS, TJW, FCM and CAW prepared and analysed bacterial fractions. TJW performed the immunofluorescence studies. YRS performed the alkaline phosphatase experiments and flow cytometry analyses. All authors contributed to the preparation of the manuscript. All authors read and approved the final manuscript.

## Supplementary Material

Additional file 1 **Figure S1.** Model of the Pet structure. Click here for file

Additional file 2 **Figure S2.** Nucleotide sequences of the *de novo* synthesised *pet* gene and heterologous DNA encoding proteins targeted for secretion. Click here for file

Additional file 3 **Figure S3.** AT-mediated accumulation of heterologous proteins in the culture medium. Click here for file

Additional file 4 **Table S1.** Mass spectrometry analysis of some recombinant protein fusions with Pet. Click here for file

Additional file 5 **Figure S4.** Surface localisation of non cleaved Pet and fusion proteins. Click here for file

Additional file 6 **Figure S5.** Identification of minimal AT module permitting secretion of heterologous proteins to the culture supernatant fraction. Click here for file

Additional file 7 **Figure S6.** Impact of conserved amino acids from the AC domain on secretion of heterologous proteins. Click here for file

Additional file 8 **Figure S7.** Comparison of the Pic and Pet SPATE proteins. Click here for file

Additional file 9 **Table S2.** Plasmids used in this study. Click here for file

Additional file 10 **Table S3.** Primers used in this study. Click here for file
