# Supplementary material for: A generalised module for the selective extracellular accumulation of recombinant proteins
Source: Microb Cell Fact. 2012 May 28;11:69. doi: 10.1186/1475-2859-11-69 (PMC3419692; doi:10.1186/1475-2859-11-69)
Supplement: Additional file 1 — Figure S1. Model of the Pet structure. [file 1475-2859-11-69-S1.pdf]

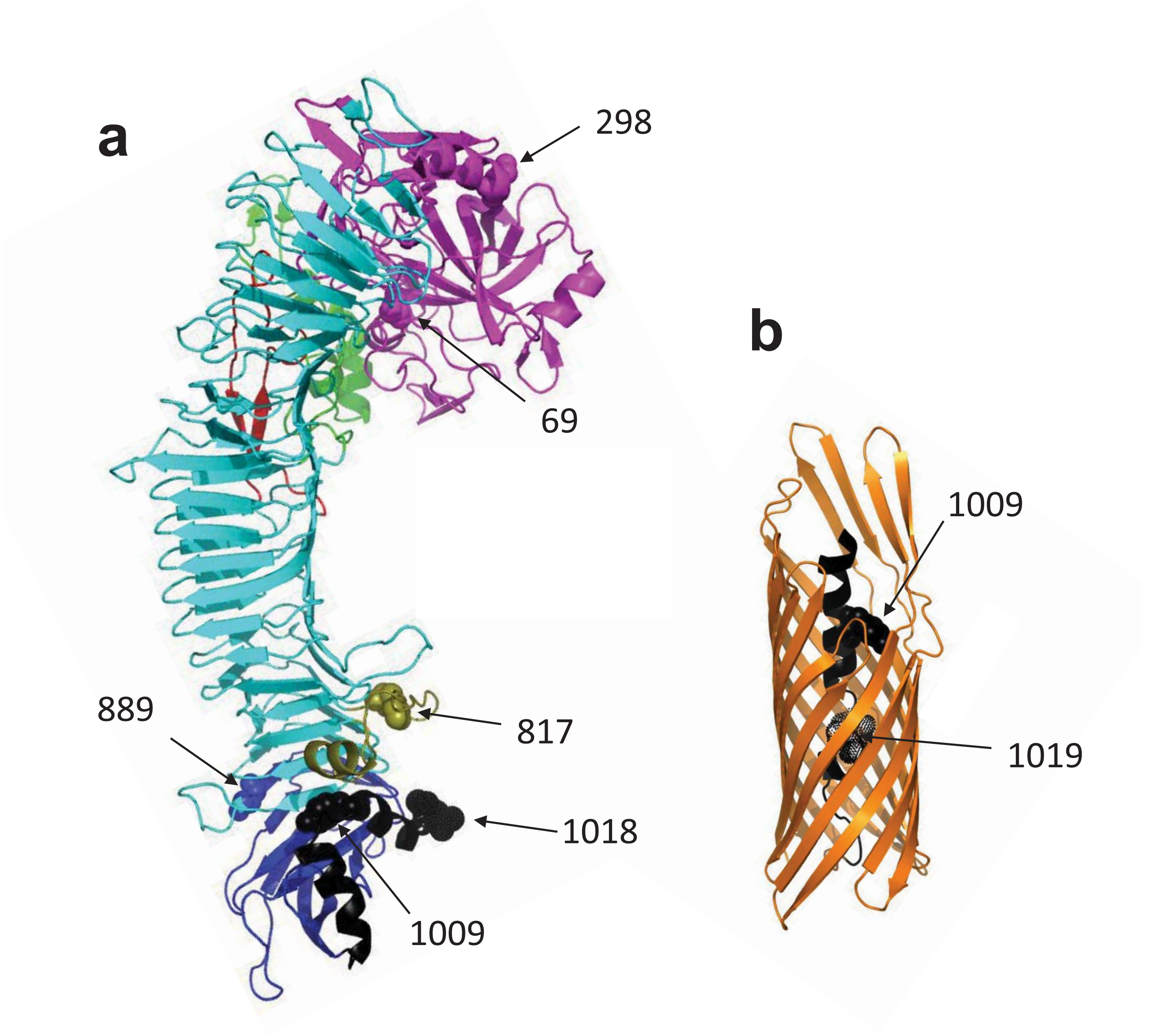


**Figure S1. Model of the Pet structure. A.** A three-dimensional model of the Pet passenger domain. Domain 1, encompassing the serine protease domain, is coloured with magenta. The β-helix is coloured with cyan. Domain 2A is coloured green. Using the Hbp nomenclature, Domains 4 and 5 are coloured red and olive, respectively. The autochaperone domain is depicted in blue. The α-helical region connecting the passenger domain to the β-barrel is represented in black. **B.** Cartoon representation of the Pet β-domain. The β-barrel is shown in orange and the α-helix, which connects to the passenger domain and contains the cleavage site, is depicted in black. The position of residues pertinent to this work are labelled and represented as spheres. Models were generated using Swiss-Model and are based on the structures resolved for the homologous EspP protein**.**
