# Supplementary material for: A generalised module for the selective extracellular accumulation of recombinant proteins
Source: Microb Cell Fact. 2012 May 28;11:69. doi: 10.1186/1475-2859-11-69 (PMC3419692; doi:10.1186/1475-2859-11-69)
Supplement: Additional file 2 — Figure S2. Nucleotide sequences of the de novo synthesised pet gene and heterologous DNA encoding proteins targeted for secretion. [file 1475-2859-11-69-S2.pdf]

A.

1 - ATGAACAAAATCTACTCTATCAAATACTCTGCGGCGACCGGCGGTCTGATCGCGGTTTCT - 60

1 - M N K I Y S I K Y S A A T G G L I A V S - 20

SacI

61 - GAGCTCGCCAAAAAAGTTATCTGCAAAACCAACCGTAAAATCTCTGCGGCGCTGCTGTCT - 120

21 - E L A K K V I C K T N R K I S A A L L S - 40

EcoRV

121 - CTGGCGGTTATCTCTTACACCAACATCATCTACGCGGCGAACATGGATATCTCTAAAGCG - 180

41 - L A V I S Y T N I I Y A A N M D I S K A - 60

BglII

181 - TGGGCGCGTGACTACCTAGATCTGGCGCAGAACAAAGGTGTTTTCCAGCCGGGTTCTACC - 240

61 - W A R D Y L D L A Q N K G V F Q P G S T - 80

241 - CACGTTAAAATCAAACTGAAAGACGGCACCGACTTCTCTTTCCCGGCGCTGCCGGTGCCG - 300

81 - H V K I K L K D G T D F S F P A L P V P - 100

301 - GACTTCTCTTCTGCGACCGCGAACGGTGCGGCGACCTCTATCGGTGGTGCTTACGCGGTT - 360

101 - D F S S A T A N G A A T S I G G A Y A V - 120

361 - ACCGTTGCTCACAACGCGAAAAACAAATCTTCTGCGAACTACCAGACCTACGGTTCTACC - 420

121 - T V A H N A K N K S S A N Y Q T Y G S T - 140

421 - CAGTACACCCAGATCAACCGTATGACCACCGGCAACGACTTCTCTATCCAGCGTCTGAAC - 480

141 - Q Y T Q I N R M T T G N D F S I Q R L N - 160

481 - AAATACGTTGTTGAAACCCGTGGTGCCGACACCTCTTTCAACTACAACGAAAACAACCAG - 540

161 - K Y V V E T R G A D T S F N Y N E N N Q - 180

ClaI

541 - AACATCATCGATAGATACGGTGTTGACGTTGGTAACGGTAAAAAAGAAATCATCGGTTTC - 600

181 - N I I D R Y G V D V G N G K K E I I G F - 200

601 - CGTGTTGGTTCTGGTAACACCACCTTCTCTGGTATCAAAACCTCTCAGACCTACCAGGCG - 660

201 - R V G S G N T T F S G I K T S Q T Y Q A - 220

661 - GACCTGCTGTCTGCGTCTCTGTTCCACATCACCAACCTGCGTGCGAACACCGTTGGTGGT - 720

221 - D L L S A S L F H I T N L R A N T V G G - 240

721 - AACAAAGTTGAATACGAAAACGACTCTTACTTCACCAACCTGACCACCAACGGTGACTCT - 780

241 - N K V E Y E N D S Y F T N L T T N G D S - 260

781 - GGTTCTGGTGTTTACGTTTTCGACAACAAAGAAGACAAATGGGTTCTGCTGGGCACCACC - 840

261 - G S G V Y V F D N K E D K W V L L G T T - 280

BstBI

841 - CACGGTATCATCGGTAACGGTAAAACCCAGAAAACCTACGTTACCCCGTTCGATTCGAAA - 900

281 - H G I I G N G K T Q K T Y V T P F D S K - 300

901 - ACCACCAACGAACTGAAACAACTGTTCATCCAGAACGTGAACATCGACAACAACACCGCG - 960

301 - T T N E L K Q L F I Q N V N I D N N T A - 320

961 - ACCATCGGTGGTGGTAAAATCACCATCGGTAACACCACCCAGGACATCGAAAAAAACAAA - 1020

321 - T I G G G K I T I G N T T Q D I E K N K - 340

1021 - AACAACCAGAACAAAGACCTGGTTTTCTCTGGTGGTGGTAAAATCTCTCTGAAAGAAAAC - 1080

341 - N N Q N K D L V F S G G G K I S L K E N - 360

1081 - CTGGACCTGGGTTACGGTGGTTTCATCTTCGACGAAAACAAAAAATACACCGTTTCTGCG - 1140

361 - L D L G Y G G F I F D E N K K Y T V S A - 380

1141 - GAAGGTAACAACAACGTGACCTTCAAAGGTGCGGGTATCGACATCGGTAAAGGTTCTACC - 1200

```
 381 - E  G  N  N  N  V  T  F  K  G  A  G  I  D  I  G  K  G  S  T   - 400

1201 - GTTGACTGGAACATCAAATACGCTTCTAACGACGCGCTGCACAAAATCGGTGAAGGTTCC - 1260
 401 - V  D  W  N  I  K  Y  A  S  N  D  A  L  H  K  I  G  E  G  S   - 420

1261 - CTGAACGTGATCCAGGCGCAGAACACCAACCTGAAAACCGGCAACGGCACCGTTATCCTG - 1320
 421 - L  N  V  I  Q  A  Q  N  T  N  L  K  T  G  N  G  T  V  I  L   - 440

                                                ApaI
1321 - GGTGCTCAGAAAACCTTCAACAACATCTACGTTGCGGGTGGGCCCGGCACCGTTCAACTG - 1380
 441 - G  A  Q  K  T  F  N  N  I  Y  V  A  G  G  P  G  T  V  Q  L   - 460

1381 - AACGCGGAAAACGCGCTGGGTGAAGGTGACTACGCGGGTATCTTCTTCACCGAAAACGGT - 1440
 461 - N  A  E  N  A  L  G  E  G  D  Y  A  G  I  F  F  T  E  N  G   - 480

1441 - GGTAAACTGGACCTGAACGGTCACAACCAGACCTTCAAAAAAATCGCGGCGACCGACTCT - 1500
 481 - G  K  L  D  L  N  G  H  N  Q  T  F  K  K  I  A  A  T  D  S   - 500

1501 - GGCACCACCATCACCAACTCTAACACCACCAAAGAATCTGTTCTGTCTGTGAACAACCAG - 1560
 501 - G  T  T  I  T  N  S  N  T  T  K  E  S  V  L  S  V  N  N  Q   - 520

1561 - AACAACTACATCTACCACGGTAACGTGGACGGTAACGTGCGTCTGGAACACCACCTGGAC - 1620
 521 - N  N  Y  I  Y  H  G  N  V  D  G  N  V  R  L  E  H  H  L  D   - 540

1621 - ACCAAACAGGACAACGCGCGTCTGATCCTGGACGGTGACATCCAGGCGAACTCTATCTCT - 1680
 541 - T  K  Q  D  N  A  R  L  I  L  D  G  D  I  Q  A  N  S  I  S   - 560

                     SpeI
1681 - ATCAAAAACGCGCCACTAGTTATGCAGGGTCACGCGACCGACCACGCGATCTTCCGTACC - 1740
 561 - I  K  N  A  P  L  V  M  Q  G  H  A  T  D  H  A  I  F  R  T   - 580

1741 - ACCAAAACCAACAACTGCCCGGAATTTCTGTGCGGTGTTGACTGGGTTACCCGTATCAAA - 1800
 581 - T  K  T  N  N  C  P  E  F  L  C  G  V  D  W  V  T  R  I  K   - 600

1801 - AACGCGGAAAACTCTGTGAACCAGAAAAACAAAACCACCTACAAATCTAACAACCAGGTT - 1860
 601 - N  A  E  N  S  V  N  Q  K  N  K  T  T  Y  K  S  N  N  Q  V   - 620

1861 - TCTGACCTGTCTCAGCCGGACTGGGAAACCCGTAAATTCCGTTTCGACAACCTGAACATC - 1920
 621 - S  D  L  S  Q  P  D  W  E  T  R  K  F  R  F  D  N  L  N  I   - 640

1921 - GAAGACTCTTCTCTGTCTATCGCGCGTAACGCGGACGTTGAAGGTAACATCCAGGCGAAA - 1980
 641 - E  D  S  S  L  S  I  A  R  N  A  D  V  E  G  N  I  Q  A  K   - 660

                                                   BsrGI
1981 - AACTCTGTTATCAACATCGGTGACAAAACCGCGTACATCGACCTGTACACTGGTAAAAAC - 2040
 661 - N  S  V  I  N  I  G  D  K  T  A  Y  I  D  L  Y  S  G  K  N   - 680

2041 - ATCACCGGCGCGGGTTTCACCTTCCGTCAGGACATCAAATCTGGTGACTCTATCGGTGAA - 2100
 681 - I  T  G  A  G  F  T  F  R  Q  D  I  K  S  G  D  S  I  G  E   - 700

2101 - TCTAAATTCACCGGCGGTATCATGGCGACCGACGGCTCCATCTCTATCGGTGACAAAGCG - 2160
 701 - S  K  F  T  G  G  I  M  A  T  D  G  S  I  S  I  G  D  K  A   - 720

                               XbaI
2161 - ATTGTTACCCTGAACACCGTTTCTTCTCTAGACCGTACCGCGCTGACCATCCACAAAGGT - 2220
 721 - I  V  T  L  N  T  V  S  S  L  D  R  T  A  L  T  I  H  K  G   - 740

2221 - GCGAACGTGACCGCGTCTTCTTCTCTGTTCACCACCTCTAACATCAAATCTGGTGGTGAC - 2280
 741 - A  N  V  T  A  S  S  S  L  F  T  T  S  N  I  K  S  G  G  D   - 760

2281 - CTGACCCTGACCGGCGCGACCGAATCTACCGGCGAAATCACCCCGTCTATGTTCTACGCG - 2340
 761 - L  T  L  T  G  A  T  E  S  T  G  E  I  T  P  S  M  F  Y  A   - 780

                               NarI
2341 - GCGGGTGGTTACGAACTGACCGAAGACGGCGCCAACTTCACCGCGAAAAACCAGGCGTCT - 2400
 781 - A  G  G  Y  E  L  T  E  D  G  A  N  F  T  A  K  N  Q  A  S   - 800
```

PstI

2401 - GTTACCGGCGACATCAAATCTGAAAAAGCGGCGAAACTGTCTTTCGGTTCTGCAGACAAA - 2460

801 - V T G D I K S E K A A K L S F G S A D K - 820

2461 - GACAACTCTGCGACCCGTTACTCTCAGTTCGCGCTGGCGATGCTGGACGGTTTCGACACC - 2520

821 - D N S A T R Y S Q F A L A M L D G F D T - 840

2521 - TCTTACCAGGGTTCTATCAAAGCGGCGCAGTCTTCTCTGGCGATGAACAACGCGCTGTGG - 2580

841 - S Y Q G S I K A A Q S S L A M N N A L W - 860

2581 - AAAGTTACCGGCAACTCTGAACTGAAAAAACTTAACTCTACCGGCTCTATGGTTCTGTTC - 2640

861 - K V T G N S E L K K L N S T G S M V L F - 880

2641 - AACGGTGGTAAAAACATCTTCAACACCCTGACCGTTGACGAACTGACCACCTCTAACTCT - 2700

881 - N G G K N I F N T L T V D E L T T S N S - 900

2701 - GCGTTCGTTATGCGTACCAACACCCAGCAGGCGGACCAACTGATCGTTAAAAACAAACTG - 2760

901 - A F V M R T N T Q Q A D Q L I V K N K L - 920

SalI

2761 - GAAGGTGCGAACAACCTGCTGCTGGTCGACTTCATCGAAAAAAAAGGTAACGACAAAAAC - 2820

921 - E G A N N L L L V D F I E K K G N D K N - 940

2821 - GGTCTGAACATCGACCTGGTTAAAGCGCCGGAAAACACCTCTAAAGACGTTTTCAAAACC - 2880

941 - G L N I D L V K A P E N T S K D V F K T - 960

2881 - GAAACCCAGACCATCGGTTTCTCTGACGTTACCCCGGAAATCAAACAGCAGGAAAAAGAC - 2940

961 - E T Q T I G F S D V T P E I K Q Q E K D - 980

2941 - GGTAAATCTGTTTGGACCCTGACCGGCTACAAAACCGTTGCGAACGCGGACGCGGCGAAA - 3000

981 - G K S V W T L T G Y K T V A N A D A A K - 1000

HpaI

3001 - AAAGCGACCTCTCTGATGTCTGGTGGTTACAAAGCGTTCCTGGCGGAAGTTAACAACCTG - 3060

1001 - K A T S L M S G G Y K A F L A E V N**N L - 1020

3061 - AACAAACGTATGGGTGACCTGCGTGACATCAACGGTGAAGCGGGTGCGTGGGCGCGTATC - 3120

1021 - N K R M G D L R D I N G E A G A W A R I - 1040

EagI

3121 - ATGTCTGGCACCGGCTCGGCCGGTGGTGGTTTCTCTGACAACTACACCCACGTTCAGGTT - 3180

1041 - M S G T G S A G G G F S D N Y T H V Q V - 1060

3181 - GGTGCGGACAACAAACACGAACTGGACGGTCTGGACCTGTTCACCGGCGTTACCATGACC - 3240

1061 - G A D N K H E L D G L D L F T G V T M T - 1080

NgoMIV

3241 - TACACCGACTCTCACGCCGGCTCTGACGCTTTCTCTGGTGAAACCAAATCTGTTGGTGCG - 3300

1081 - Y T D S H A G S D A F S G E T K S V G A - 1100

3301 - GGTCTGTACGCTTCTGCGATGTTTGAATCTGGTGCGTACATCGACCTGATCGGTAAATAC - 3360

1101 - G L Y A S A M F E S G A Y I D L I G K Y - 1120

KpnI

3361 - GTTCACCACGACAACGAATACACCGCGACCTTCGCGGGTCTGGGTACCCGTGACTACTCT - 3420

1121 - V H H D N E Y T A T F A G L G T R D Y S - 1140

3421 - TCTCACTCTTGGTACGCGGGTGCGGAAGTTGGTTACCGTTACCACGTTACCGACTCTGCG - 3480

1141 - S H S W Y A G A E V G Y R Y H V T D S A - 1160

3481 - TGGATCGAACCGCAGGCGGAACTGGTTTACGGTGCGGTTTCTGGTAAACAGTTCTCTTGG - 3540

1161 - W I E P Q A E L V Y G A V S G K Q F S W - 1180

3541 - AAAGACCAGGGTATGAACCTGACCATGAAAGACAAAGACTTCAACCCGCTGATCGGTCGT - 3600

1181 - K D Q G M N L T M K D K D F N P L I G R - 1200

AatII

```
3601 - ACCGGCGTTGACGTCGGTAAATCTTTCTCTGGTAAAGACTGGAAAGTTACCGCGCGTGCG - 3660
1201 - T  G  V  D  V  G  K  S  F  S  G  K  D  W  K  V  T  A  R  A   - 1220

3661 - GGTCTGGGTTACCAGTTCGACCTGTTCGCTAACGGTGAAACCGTTCTGCGTGACGCTTCT - 3720
1221 - G  L  G  Y  Q  F  D  L  F  A  N  G  E  T  V  L  R  D  A  S   - 1240

3721 - GGTGAAAAACGTATCAAAGGTGAAAAAGACGGTCGTATGCTGATGAACGTGGGTCTGAAC - 3780
1241 - G  E  K  R  I  K  G  E  K  D  G  R  M  L  M  N  V  G  L  N   - 1260
                                         EcoRI
3781 - GCGGAAATCCGTGACAACGTGCGTTTCGGTCTGGAATTCGAGAAATCTGCGTTCGGTAAA - 3840
1261 - A  E  I  R  D  N  V  R  F  G  L  E  F  E  K  S  A  F  G  K   - 1280

3841 - TACAACGTGGACAACGCGATCAACGCGAACTTCCGTTACTCTTTCTGA - 3888
1281 - Y  N  V  D  N  A  I  N  A  N  F  R  Y  S  F  *   - 1300
```

B.

Pmp17

```
  1 - AACGACGCGCAGACCGCGCTGACCCCGTCTGACTCTTACAACGGTAACGTTACCTCTGAA - 60
  1 - N  D  A  Q  T  A  L  T  P  S  D  S  Y  N  G  N  V  T  S  E   - 20

 61 - GAATTCCAGGTTAAAGAAACCTCTTCTGGTACCACCTACACCTGCGAAGGTAACGTTTGC - 120
 21 - E  F  Q  V  K  E  T  S  S  G  T  T  Y  T  C  E  G  N  V  C   - 40

121 - ATCTCTTTCGCGGGTAAAGACTCTGGTCTGAAAAAATCTTGCTTCTCTGCGACCGACAAC - 180
 41 - I  S  F  A  G  K  D  S  G  L  K  K  S  C  F  S  A  T  D  N   - 60

181 - CTGACCTTCCTGGGTAACGGTTACACCCTGTGCTTCGACAACATCACCACCACCGCGTCT - 240
 61 - L  T  F  L  G  N  G  Y  T  L  C  F  D  N  I  T  T  T  A  S   - 80

241 - AACCCGGGTGCGATCAACGTTCAGGGTCAGGGTAAAACCCTGGGTATCTCTGGTTTCTCT - 300
 81 - N  P  G  A  I  N  V  Q  G  Q  G  K  T  L  G  I  S  G  F  S   - 100

301 - CTGTTCTCTTGCGCGTACTGCCCGCCGGGTACCACCGGTTACGGTGCGATCCAGACCAAA - 360
101 - L  F  S  C  A  Y  C  P  P  G  T  T  G  Y  G  A  I  Q  T  K   - 120

361 - GGTAACACCACCCTGAAAGACAACTCTTCTCTGGTTTTCCACAAAAACTGCTCTACCGCG - 420
121 - G  N  T  T  L  K  D  N  S  S  L  V  F  H  K  N  C  S  T  A   - 140

421 - GAAGGTGGTGCGATCCAGTGCAAAGGTTCTTCTGACGCGGAACTGAAAATCGAAAACAAC - 480
141 - E  G  G  A  I  Q  C  K  G  S  S  D  A  E  L  K  I  E  N  N   - 160

481 - CAGAACCTGGTTTTCTCTGAAAACTCTTCTACCTCTAAAGGTGGTGCGATCTACGCGGAC - 540
161 - Q  N  L  V  F  S  E  N  S  S  T  S  K  G  G  A  I  Y  A  D   - 180

541 - AAACTGACCATCGTTTCTGGTGGTCCGACCCTGTTCTCTAACAACTCTGTTTCTAACGGT - 600
181 - K  L  T  I  V  S  G  G  P  T  L  F  S  N  N  S  V  S  N  G   - 200

601 - TCTTCTCCGAAAGGTGGTGCGATCTCTATCAAAGACTCTTCTGGTGAATGCTCTCTGACC - 660
201 - S  S  P  K  G  G  A  I  S  I  K  D  S  S  G  E  C  S  L  T   - 220

661 - GCGGACCTGGGTGACATCACCTTCGACGGTAACAAAATCATCAAAACCTCTGGTGGTTCT - 720
221 - A  D  L  G  D  I  T  F  D  G  N  K  I  I  K  T  S  G  G  S   - 240

721 - TCTACCGTTACCCGTAACTCTATCGACCTGGGTACCGGTAAATTCACCAAACTGCGTGCG - 780
241 - S  T  V  T  R  N  S  I  D  L  G  T  G  K  F  T  K  L  R  A   - 260
```

781 - AAAGACGGTTTCGGTATCTTCTTCTACGACCCGATCACCGGTGGTGGTTCTGACGAACTG - 840
261 - K D G F G I F F Y D P I T G G G S D E L - 280

841 - AACATCAACAAAAAAGAAACCGTTGACTACACCGGTAAAATCGTTTTCTCTGGTGAAAAA - 900
281 - N I N K K E T V D Y T G K I V F S G E K - 300

901 - CTGTCTGACGAAGAAAAAGCGCGTGCGGAAAACCTGGCGTCTACCTTCAACCAGCCGATC - 960
301 - L S D E E K A R A E N L A S T F N Q P I - 320

961 - ACCCTGTCTGCGGGTTCTCTGGTTCTGAAAGACGGTGTTTCTGTTACCGCGAAACAGGTT - 1020
321 - T L S A G S L V L K D G V S V T A K Q V - 340

1021 - ACCCAGGAAGCGGGTTCTACCGTTGTTATGGACCTGGGTACCACCCTGCAAACCCCGTCT - 1080
341 - T Q E A G S T V V M D L G T T L Q T P S - 360

1081 - TCTGGTGGTGAAACCATCACCCTGACCAACCTGGACATCAACATCGCGTCTCTGGGTGGT - 1140
361 - S G G E T I T L T N L D I N I A S L G G - 380

1141 - GGTGGTGGTACCTCTCCGGCGAAACTGGCG - 1170
381 - G G G T S P A K L A

YapA

1 - GTTTCTCAGATCGCGACCACCGACACCCCGGCGGTTACCCCGATCAAATCTACCCTGACC - 60
1 - V S Q I A T T D T P A V T P I K S T L T - 20

61 - GGTCCGTTTGAGCGTAACTCTGCGGGTACCTCTTTCGGTTCTAACGTTGACGTTGACGTT - 120
21 - G P F E R N S A G T S F G S N V D V D V - 40

121 - ATCGACAACACCTCTACCGCGACCCGTGTTATCGCGGAAACCACCCCGGAAGCGGAATCT - 180
41 - I D N T S T A T R V I A E T T P E A E S - 60

181 - ACCATCGGTGAAGCGACCGGTCAGGAAGGTGGTAACGCGACCGCGGTTATCCCGCCGACC - 240
61 - T I G E A T G Q E G G N A T A V I P P T - 80

241 - ACCACCCCGTCTGAACAGGAAATCACCGAACCGGAACAGCCGGGTCTGCTGGACAAAATC - 300
81 - T T P S E Q E I T E P E Q P G L L D K I - 100

301 - AAAGACCTGCTGGGTCTGGGTGAAATCACCCAGGAACAGGCGGACGCGCTGGAAAAAAAC - 360
101 - K D L L G L G E I T Q E Q A D A L E K N - 120

361 - GTTAAAACCAAAGTTGAAAAAGTTGACGCGCAGACCGCGGCGAAACTGGCGCTGGAATCT - 420
121 - V K T K V E K V D A Q T A A K L A L E S - 140

421 - GCGCAGGCGGAAGCGCAGAAAGCGGCGGAAGACGCGCTGTACCTGAAAACCGAAAACGTT - 480
141 - A Q A E A Q K A A E D A L Y L K T E N V - 160

481 - TCTTACCAGGCGTTCGCGCAGACCGAAGAAAAAATCAAAAAAGAAGCGGACGAAGCGAAA - 540
161 - S Y Q A F A Q T E E K I K K E A D E A K - 180

541 - AAAAAACAGGACAAAACCAAAGAAGACGCGATCAAAGCGGTTAAAGTTAACAACACCCCG - 600
181 - K K Q D K T K E D A I K A V K V N N T P - 200

601 - CTGGTTCCGGGTGACAAAGACATCGCGGAAAAAGTTACCAAAGCGGTTACCGACACCACC - 660

201 - L V P G D K D I A E K V T K A V T D T T - 220

661 - AAAGTTCAGGGTGAAAAAGCGGTTACCCTGGCGACCAAAATCACCGACGCGAAAGTTGCG - 720
221 - K V Q G E K A V T L A T K I T D A K V A - 240

721 - CAGGAAAAAAAAGACGCGAACACCGAAGCGCTGGCGGAAATCGACGGTCGTCTGATCTCT - 780
241 - Q E K K D A N T E A L A E I D G R L I S - 260

781 - GTTTCTAACGCGCTGATCCAGGCGACCGGTACCGACAAAGGTCCGCTGGACCAGAAACTG - 840
261 - V S N A L I Q A T G T D K G P L D Q K L - 280

841 - AAAGAAGCGCAGCAGGCGAAAACCGAACAGGACGGTAAAGAACTGGCGTCTGGTGGTTAC - 900
281 - K E A Q Q A K T E Q D G K E L A S G G Y - 300

901 - AAAGAACTGTTTGAGGAAGACAAAAAAACCTCTGGTTACTTCGGTATCGCGGAAAACGAC - 960
301 - K E L F E E D K K T S G Y F G I A E N D - 320

961 - AACGGTTCTGGTCAGCAGGAAAAACTGGCGGAAGCGAAAAAAAACCGTGACGCGTACAAC - 1020
321 - N G S G Q Q E K L A E A K K N R D A Y N - 340

1021 - AAAGCGGCGAAAAAAGAACTGGACGCGATCGCGAAAGCGCAGAAAGCGGTTGAAGCGATC - 1080
341 - K A A K K E L D A I A K A Q K A V E A I - 360

1081 - GACGCGCAGATCGTTAAACTGAAAAAAGACAAAGGTGACATCGAACAGGAACAGTCTACC - 1140
361 - D A Q I V K L K K D K G D I E Q E Q S T - 380

1141 - GAAAAAGGTAAAACCGGTGGTCTGGACATCGCGCTGTCTGGTGCGAACGACGCGAAAGAC - 1200
381 - E K G K T G G L D I A L S G A N D A K D - 400

1201 - GCGGCGCAGGGTGAATTCGACACCGCGAAAAACGCGGCGGAACTGGCGGAAACCGCGGCG - 1260
401 - A A Q G E F D T A K N A A E L A E T A A - 420

1261 - AAAGCGATCGAAGCGGCGAAAATCACCGACAAAGCGGTTGAAGACGCGACCGCGGCGTAC - 1320
421 - K A I E A A K I T D K A V E D A T A A Y - 440

1321 - AAAGAAGCGGCGGACAAAGCGGAACAGACCAAAACCGCGCTGGAAGCGGCGGAAAAAGCG - 1380
441 - K E A A D K A E Q T K T A L E A A E K A - 460

1381 - AAAGAAGACGCGGACAAACTGGTTGTTACCAACACCGGTCTGCTGAACGACGCGGACCAG - 1440
461 - K E D A D K L V V T N T G L L N D A D Q - 480

1441 - GCGCTGGAACAGCTGGTTACCGCGCAGAACAACGCGCAGCCGACCCTGGACCTGCCGGCG - 1500
481 - A L E Q L V T A Q N N A Q P T L D L P A - 500

1501 - ATCGACGTTACCATCGCGCCGGCGAAAACCCAGGACGTTATCGAAGGTACCTCTGCGATC - 1560
501 - I D V T I A P A K T Q D V I E G T S A I - 520

1561 - GCGACCCAGGTTGCGGGTGGTACCCAGAACGTTGCGAAAGGTGGTAAAGCGATCGACTCT - 1620
521 - A T Q V A G G T Q N V A K G G K A I D S - 540

1621 - GTTATCACCAAAGACGGTATCGTTAACCTGGCGGCGGGTGCGAACGCGAAAGGTACCGAA - 1680
541 - V I T K D G I V N L A A G A N A K G T E - 560

1681 - GTTACCAAAGGTACCCTGAACAACAACGGTGGTGTTGACACCGACACCGTTGTTTCTACC - 1740
561 - V T K G T L N N N G G V D T D T V V S T - 580

1741 - GAAGGTAAACTGGTTCTGACCGGTGGTTCTGAAACCGCGATCGCGACCTCTACCGGTGCG - 1800
581 - E G K L V L T G G S E T A I A T S T G A - 600

1801 - AAAGTTGCGGAAGGTGGTGTTGTTACCGCGGGTGACCACTCTGTTATCGAAAAAATGATC - 1860
601 - K V A E G G V V T A G D H S V I E K M I - 620

1861 - TCTTCTGGTAACGTTACCGCGTCTGGTAACAACACCATCGTTCGTGACACCACCATCAAC - 1920
621 - S S G N V T A S G N N T I V R D T T I N - 640

1921 - GACGGTAAACTGTCTCTGGCGGGTACCGCGACCGCGAACAACACCACCTTCAACGGTGGT - 1980
641 - D G K L S L A G T A T A N N T T F N G G - 660

1981 - ATCTTCTCTGTTGAAGGTGACACCGCGGCGACCAAAACCAACATGACCGGTGGTAAATTC - 2040
661 - I F S V E G D T A A T K T N M T G G K F - 680

2041 - GCGGTTACCGGTAACGCGAAAATCGAAGAAACCGTTGTTTCTGCGGGTGACGTTTCTCTG - 2100
681 - A V T G N A K I E E T V V S A G D V S L - 700

2101 - GCGGACAAAGCGACCGCGAACAACACCACCCTGACCGGTGGTACCTTCGCGGCGGCGGGT - 2160
701 - A D K A T A N N T T L T G G T F A A A G - 720

2161 - GACACCGCGGTTTCTGCGACCAACATGACCGGTGGTACCTTCACCGTTGCGGGTGACACC - 2220
721 - D T A V S A T N M T G G T F T V A G D T - 740

2221 - GCGGCGACCAAAACCAAAATGACCGGTGGTGAATTCGCGGTTACCGGTAACGCGAAAATC - 2280
741 - A A T K T K M T G G E F A V T G N A K I - 760

2281 - GAAGACACCGTTCTGAACGCGTCTGACTTCTCTCTGGCGGACAAAGCGACCGCGAACAAC - 2340
761 - E D T V L N A S D F S L A D K A T A N N - 780

2341 - ACCACCCTGACCGACGGTACCTTCACCGTTGCGGGTGACGCGGCGGTTACCGCGACCAAC - 2400
781 - T T L T D G T F T V A G D A A V T A T N - 800

2401 - ATGTCTGGTGGTAAATTCGCGGTTAAAGGTAAAGCGAAAATCAAAGACACCCAGCTGTCT - 2460
801 - M S G G K F A V K G K A K I K D T Q L S - 820

2461 - GCGGGTAACTTCACCCTGGCGGAAAACGCGACCGCGAACGACACCACCCTGAACGGTGGT - 2520
821 - A G N F T L A E N A T A N D T T L N G G - 840

2521 - AAATTCGACGTTTCTAACGAAGCGACCGCGACCAACACCACCATCAACAACGGTCTGTTC - 2580
841 - K F D V S N E A T A T N T T I N N G L F - 860

2581 - ACCCTGAAAGACGGTGCGCACGCGGACTCTACCACCGTTAACTCTGGTACCTTCGTTATG - 2640
861 - T L K D G A H A D S T T V N S G T F V M - 880

2641 - GCGGACCAGTCTACCGCGAACGGTATCCAGCTGGTTGACTCTGCGTTCACCCTGGCGTCT - 2700
881 - A D Q S T A N G I Q L V D S A F T L A S - 900

2701 - GGTGCGAAAGCGTCTGGTATCACCAAACTGACCGGTGGTCAGGCGCAGGTTGCGGGTTCT - 2760
901 - G A K A S G I T K L T G G Q A Q V A G S - 920

2761 - CTGGAATCTCTGTCTCTGACCGGTGGTCGTGCGGACTTCGCGAACTCTGCGAAAGCGTCT - 2820
921 - L E S L S L T G G R A D F A N S A K A S - 940

2821 - GGTCTGCTGGACATCTCTGCGGACTCTCAGATCATTATGAACCGTGGTGCGGACACCGCG - 2880
941 - G L L D I S A D S Q I I M N R G A D T A - 960

2881 - CAGGCGAACCTGAACCTGGCGGGTCGTCTGGAACTGCTGGCGTCTGACGTTGCGCAGGCG - 2940
961 - Q A N L N L A G R L E L L A S D V A Q A - 980

2941 - GTTGCGCAGCCGGTTGCGCGTGCGGCGATGGAACTGTCTAACGCGCGTGCGGTTATGCCG - 3000
981 - V A Q P V A R A A M E L S N A R A V M P - 1000

3001 - GCGCCGGCGATGCCGGTTCCGGCGGCGGCGCCGGTTGCGCACTTCGCGCTGAACGACGTT - 3060
1001 - A P A M P V P A A A P V A H F A L N D V - 1020

```
3061 - GTTATGACCGGTGGTACCGTTGACATGTCTAACGCG - 3096
1021 - V  M  T  G  G  T  V  D  M  S  N  A
```

SapA

```
   1 - ACCACCTATGATACCTGGACCTATTATGATAATCCGACCACCGCGCTGGATTGGAACAAC - 60
   1 - T  T  Y  D  T  W  T  Y  Y  D  N  P  T  T  A  L  D  W  N  N   - 20

  61 - ATGGATGCCGCGGGCACCGTGGATGGCAACTATGTGAACTATAGCGGCTTTGTGTATTAT - 120
  21 - M  D  A  A  G  T  V  D  G  N  Y  V  N  Y  S  G  F  V  Y  Y   - 40

 121 - AACAACGCGAACGGCGATTTTGATCAGACCTTTAACGGCGATACCGTGAACGGCACCATT - 180
  41 - N  N  A  N  G  D  F  D  Q  T  F  N  G  D  T  V  N  G  T  I   - 60

 181 - AGCACCTATTATCTGAACCATGATTATAACGATGCGACCTCGAACGAACTGAACATTAGC - 240
  61 - S  T  Y  Y  L  N  H  D  Y  N  D  A  T  S  N  E  L  N  I  S   - 80

 241 - AACAGCGTGATTCATGGCAGCATTACCAGCATGCTGCCGATTGGCTACTACGATCGCTTT - 300
  81 - N  S  V  I  H  G  S  I  T  S  M  L  P  I  G  Y  Y  D  R  F   - 100

 301 - GATACCCTGGATCATGATGGCTATAGCGAATATTATCGCTTTAACAACGGCACGGATACC - 360
 101 - D  T  L  D  H  D  G  Y  S  E  Y  Y  R  F  N  N  G  T  D  T   - 120

 361 - GTCGATGGCAATTGGTATGATGGCGATGTGTTTACCCTGAACATTGCGAACAGCACCATC - 420
 121 - V  D  G  N  W  Y  D  G  D  V  F  T  L  N  I  A  N  S  T  I   - 140

 421 - GATGATGATTATGAAGCGTTTTATTTTACCGATAGCTATAAAGATGGCGATGTCACCAAA - 480
 141 - D  D  D  Y  E  A  F  Y  F  T  D  S  Y  K  D  G  D  V  T  K   - 160

 481 - TATACCAACGAAACCTTTGATGTGTCCGAAGGCGTGGCGGTGAACCTGGATGTGGAAAGC - 540
 161 - Y  T  N  E  T  F  D  V  S  E  G  V  A  V  N  L  D  V  E  S   - 180

 541 - AACATTAACATCAGCAACAACAGCCGCGTGGCGGGCATTGCGCTGTCCCAGGGCAACACC - 600
 181 - N  I  N  I  S  N  N  S  R  V  A  G  I  A  L  S  Q  G  N  T   - 200

 601 - TATAACAACACCTATACCACCGAAAGCCATAACTGGGATAACAACATCAACGTGTTTGAT - 660
 201 - Y  N  N  T  Y  T  T  E  S  H  N  W  D  N  N  I  N  V  F  D   - 220

 661 - AGCACCGTGACCAGCGGCAGCGATTATATTCTGGATAGCGCGTATACCACCGATACCGGC - 720
 221 - S  T  V  T  S  G  S  D  Y  I  L  D  S  A  Y  T  T  D  T  G   - 240

 721 - ACCTTTGGCACCGGCCATTTTGGCAACAGCGATGAACCGAGCGATTATACCGGTGCGGGT - 780
 241 - T  F  G  T  G  H  F  G  N  S  D  E  P  S  D  Y  T  G  A  G   - 260

 781 - GATGTCGCGCTGTCCTTTACCGATGATAACGGCGCGAGCGATTATGCGATGAAAAACAAC - 840
 261 - D  V  A  L  S  F  T  D  D  N  G  A  S  D  Y  A  M  K  N  N   - 280

 841 - GTGTATTTTAGCAACAGCACCCTGATGGGCGATGTGAAATTTACCAGCAACTGGAACGCG - 900
 281 - V  Y  F  S  N  S  T  L  M  G  D  V  K  F  T  S  N  W  N  A   - 300

 901 - AACTTTGATGCGGATGGCGATGATACCAATGGCGATGGCGTCCCGGATACCAACCATGGC - 960
 301 - N  F  D  A  D  G  D  D  T  N  G  D  G  V  P  D  T  N  H  G   - 320

 961 - TGGGCGGATGATGGCCTGAACGTGGATGAACTGAACCTGACCCTGGATAACGGCAGCAAA - 1020
 321 - W  A  D  D  G  L  N  V  D  E  L  N  L  T  L  D  N  G  S  K   - 340

1021 - TGGGTGGGCCAGGCGACCTATACCGTGGATACCACGAGCCGCATGTATGATGTGGAAACC - 1080
 341 - W  V  G  Q  A  T  Y  T  V  D  T  T  S  R  M  Y  D  V  E  T   - 360

1081 - AATAGCCTGACCCCGGGTGCGACGCTGGAAGATAACGCGTGGAACCGCATTGTGGGCAAC - 1140
```

```
 361 - N  S  L  T  P  G  A  T  L  E  D  N  A  W  N  R  I  V  G  N   - 380

1141 - GAAGTGTTTCAGAGCGGCGTGTTTAACGTGACGCTGAACAATGGCTCGGAATGGGATACC - 1200
 381 - E  V  F  Q  S  G  V  F  N  V  T  L  N  N  G  S  E  W  D  T   - 400

1201 - GTGGGCGATAGCACGGTGGATACCCTGGCGGTGAACAACGGCAGCCAGGTGAACGTGAGC - 1260
 401 - V  G  D  S  T  V  D  T  L  A  V  N  N  G  S  Q  V  N  V  S   - 420

1261 - AACAGCGATCTGACCAGCGATACCATTGATCTGACCAACGGCAGCAGCCTGAATATTGGC - 1320
 421 - N  S  D  L  T  S  D  T  I  D  L  T  N  G  S  S  L  N  I  G   - 440

1321 - GAAGGCGGCTATGTGGATACCGATCATCTGACCATTGATAGCTATAGCACCGTGGGCCTG - 1380
 441 - E  G  G  Y  V  D  T  D  H  L  T  I  D  S  Y  S  T  V  G  L   - 460

1381 - ACCGAAAGCACCGGCTGGTCCACCTATAGCAACCTGTATGCGAACACCATTACCGTCACC - 1440
 461 - T  E  S  T  G  W  S  T  Y  S  N  L  Y  A  N  T  I  T  V  T   - 480

1441 - AATGGCGGCGTGCTGGATGTGAACGTGGGCCAGTTTGATACCGAAGTGTTTAGCACCGAT - 1500
 481 - N  G  G  V  L  D  V  N  V  G  Q  F  D  T  E  V  F  S  T  D   - 500

1501 - AAACTGGAACTGACGAGCGGCAACACCGCGGATCATAACGGCAACGTGGTGAGCGGCGTC - 1560
 501 - K  L  E  L  T  S  G  N  T  A  D  H  N  G  N  V  V  S  G  V   - 520

1561 - TTTAATATTCACAGCAGCGATTATGTGCTGAACGCGGATCTGGTGAACGATCGCACCTGG - 1620
 521 - F  N  I  H  S  S  D  Y  V  L  N  A  D  L  V  N  D  R  T  W   - 540

1621 - GATACCACGCAGGCGAACTATGGCTATGGCACCATTGCGATGAACAGCGATGGC - 1674
 541 - D  T  T  Q  A  N  Y  G  Y  G  T  I  A  M  N  S  D  G
```

Pertactin

```
   1 - GACTGGAACAACCAGTCTATCGTTAAAACCGGTGAACGTCAGCACGGTATCCACATCCAG - 60
   1 - D  W  N  N  Q  S  I  V  K  T  G  E  R  Q  H  G  I  H  I  Q   - 20

  61 - GGTTCTGATCCGGGTGGTGTTCGTACCGCGTCTGGTACCACCATCAAAGTTTCTGGTCGT - 120
  21 - G  S  D  P  G  G  V  R  T  A  S  G  T  T  I  K  V  S  G  R   - 40

 121 - CAGGCGCAGGGTATCCTGCTGGAAAACCCGGCGGCGGAACTGCAGTTCCGTAACGGTTCT - 180
  41 - Q  A  Q  G  I  L  L  E  N  P  A  A  E  L  Q  F  R  N  G  S   - 60

 181 - GTTACCTCTTCTGGTCAACTGTCTGACGACGGTATCCGTCGTTTCCTGGGTACCGTTACC - 240
  61 - V  T  S  S  G  Q  L  S  D  D  G  I  R  R  F  L  G  T  V  T   - 80

 241 - GTTAAAGCGGGTAAACTGGTTGCGGACCACGCGACCCTGGCGAACGTTGGTGACACCTGG - 300
  81 - V  K  A  G  K  L  V  A  D  H  A  T  L  A  N  V  G  D  T  W   - 100

 301 - GACGACGACGGTATCGCGCTGTACGTTGCGGGTGAACAGGCGCAGGCGTCTATCGCGGAC - 360
 101 - D  D  D  G  I  A  L  Y  V  A  G  E  Q  A  Q  A  S  I  A  D   - 120

 361 - TCTACCCTGCAGGGTGCGGGTGGTGTTCAGATCGAACGTGGTGCGAACGTTACCGTTCAG - 420
 121 - S  T  L  Q  G  A  G  G  V  Q  I  E  R  G  A  N  V  T  V  Q   - 140

 421 - CGTTCTGCTATCGTTGACGGTGGTCTGCACATCGGTGCGCTGCAGTCTCTGCAGCCGGAA - 480
 141 - R  S  A  I  V  D  G  G  L  H  I  G  A  L  Q  S  L  Q  P  E   - 160

 481 - GACCTGCCGCCGTCTCGTGTTGTTCTGCGTGACACCAACGTTACCGCTGTTCCGGCGTCT - 540
 161 - D  L  P  P  S  R  V  V  L  R  D  T  N  V  T  A  V  P  A  S   - 180

 541 - GGTGCTCCGGCGGCGGTTTCTGTTCTGGGTGCGTCTGAACTGACCCTGGACGGTGGTCAC - 600
 181 - G  A  P  A  A  V  S  V  L  G  A  S  E  L  T  L  D  G  G  H   - 200
```

```
 601 - ATCACCGGTGGTCGTGCGGCGGGTGTTGCGGCGATGCAGGGTGCGGTTGTTCACCTGCAG - 660
 201 - I  T  G  G  R  A  A  G  V  A  A  M  Q  G  A  V  V  H  L  Q   - 220

 661 - CGTGCGACCATCCGTCGTGGTGACGCTCCGGCGGGTGGTGCGGTTCCGGGTGGTGCGGTT - 720
 221 - R  A  T  I  R  R  G  D  A  P  A  G  G  A  V  P  G  G  A  V   - 240

 721 - CCGGGTGGTGCGGTTCCGGGTGGTTTCGGTCCGGGTGGTTTCGGTCCGGTTCTGGACGGT - 780
 241 - P  G  G  A  V  P  G  G  F  G  P  G  G  F  G  P  V  L  D  G   - 260

 781 - TGGTACGGTGTTGACGTTTCTGGTTCTTCTGTTGAACTGGCGCAGTCTATCGTTGAAGCG - 840
 261 - W  Y  G  V  D  V  S  G  S  S  V  E  L  A  Q  S  I  V  E  A   - 280

 841 - CCGGAACTGGGTGCGGCGATCCGTGTTGGTCGTGGTGCGCGTGTTACCGTTTCTGGTGGT - 900
 281 - P  E  L  G  A  A  I  R  V  G  R  G  A  R  V  T  V  S  G  G   - 300

 901 - TCTCTGTCTGCGCCGCACGGTAACGTTATCGAAACCGGTGGTGCGCGTCGTTTCGCGCCG - 960
 301 - S  L  S  A  P  H  G  N  V  I  E  T  G  G  A  R  R  F  A  P   - 320

 961 - CAGGCTGCGCCGCTGTCTATCACCCTGCAGGCGGGCGCGCACGCGCAGGGTAAAGCTCTG - 1020
 321 - Q  A  A  P  L  S  I  T  L  Q  A  G  A  H  A  Q  G  K  A  L   - 340

1021 - CTGTACCGTGTTCTGCCGGAACCGGTTAAACTGACCCTGACCGGTGGTGCGGACGCGCAG - 1080
 341 - L  Y  R  V  L  P  E  P  V  K  L  T  L  T  G  G  A  D  A  Q   - 360

1081 - GGTGACATCGTTGCGACCGAACTGCCGTCTATCCCGGCTACCTCTATCGGTCCGCTGGAC - 1140
 361 - G  D  I  V  A  T  E  L  P  S  I  P  A  T  S  I  G  P  L  D   - 380

1141 - GTTGCGCTGGCGTCTCAGGCGCGTTGGACCGGTGCGACCCGTGCGGTTGACTCTCTGTCT - 1200
 381 - V  A  L  A  S  Q  A  R  W  T  G  A  T  R  A  V  D  S  L  S   - 400

1201 - ATCGACAACGCGACCTGGGTTATGACCGACAACTCTAACGTTGGTGCGCTGCGTCTGGCG - 1260
 401 - I  D  N  A  T  W  V  M  T  D  N  S  N  V  G  A  L  R  L  A   - 420

1261 - TCTGACGGTTCTGTTGACTTCCAGCAACCGGCGGAAGCGGGTCGTTTCAAA - 1311
 421 - S  D  G  S  V  D  F  Q  Q  P  A  E  A  G  R  F  K
```

mCherry

```
   1 - ATGGTTTCTAAAGGTGAAGAAGACAACATGGCTATCATCAAAGAATTTATGCGTTTCAAA - 60
   1 - M  V  S  K  G  E  E  D  N  M  A  I  I  K  E  F  M  R  F  K   - 20

  61 - GTTCACATGGAAGGTTCTGTGAACGGTCACGAATTTGAAATCGAAGGTGAAGGTGAAGGT - 120
  21 - V  H  M  E  G  S  V  N  G  H  E  F  E  I  E  G  E  G  E  G   - 40

 121 - CGTCCGTATGAAGGCACCCAGACCGCTAAACTGAAAGTTACCAAAGGTGGTCCGCTGCCG - 180
  41 - R  P  Y  E  G  T  Q  T  A  K  L  K  V  T  K  G  G  P  L  P   - 60

 181 - TTCGCTTGGGACATCCTGTCTCCGCAGTTCATGTACGGTTCTAAAGCGTATGTTAAACAC - 240
  61 - F  A  W  D  I  L  S  P  Q  F  M  Y  G  S  K  A  Y  V  K  H   - 80

 241 - CCGGCTGACATCCCGGACTACCTGAAACTGTCTTTCCCGGAAGGTTTCAAATGGGAACGT - 300
  81 - P  A  D  I  P  D  Y  L  K  L  S  F  P  E  G  F  K  W  E  R   - 100

 301 - GTTATGAACTTTGAAGACGGTGGTGTTGTTACCGTTACCCAGGACTCTTCTCTGCAAGAC - 360
 101 - V  M  N  F  E  D  G  G  V  V  T  V  T  Q  D  S  S  L  Q  D   - 120

 361 - GGTGAATTTATCTACAAAGTTAAACTGCGTGGCACCAACTTCCCGTCTGACGGTCCGGTT - 420
 121 - G  E  F  I  Y  K  V  K  L  R  G  T  N  F  P  S  D  G  P  V   - 140
```

```
421 - ATGCAGAAAAAAACGATGGGTTGGGAAGCGTCTTCTGAACGTATGTACCCGGAAGACGGT - 480
141 - M  Q  K  K  T  M  G  W  E  A  S  S  E  R  M  Y  P  E  D  G  - 160

481 - GCTCTGAAAGGTGAAATCAAACAGCGTCTGAAACTGAAAGACGGTGGTCACTACGACGCT - 540
161 - A  L  K  G  E  I  K  Q  R  L  K  L  K  D  G  G  H  Y  D  A  - 180

541 - GAAGTTAAAACCACCTACAAAGCTAAAAAGCCGGTTCAACTGCCGGGTGCTTACAACGTG - 600
181 - E  V  K  T  T  Y  K  A  K  K  P  V  Q  L  P  G  A  Y  N  V  - 200

601 - AACATCAAACTGGACATCACCTCTCACAACGAAGACTACACCATCGTTGAACAGTACGAA - 660
201 - N  I  K  L  D  I  T  S  H  N  E  D  Y  T  I  V  E  Q  Y  E  - 220

661 - CGTGCTGAAGGTCGTCACTCTACCGGCGGTATGGACGAACTGTATAAA - 708
221 - R  A  E  G  R  H  S  T  G  G  M  D  E  L  Y  K
```

ESAT-6

```
  1 - ATGACCGAACAGCAGTGGAACTTCGCTGGTATCGAAGCTGCTGCTTCTGCTATCCAGGGT - 60
  1 - M  T  E  Q  Q  W  N  F  A  G  I  E  A  A  A  S  A  I  Q  G  - 20

 61 - AACGTAACCTCTATCCACTCTCTGCTGGACGAAGGTAAACAGTCTCTGACCAAACTGGCT - 120
 21 - N  V  T  S  I  H  S  L  L  D  E  G  K  Q  S  L  T  K  L  A  - 40

121 - GCTGCTTGGGGTGGTTCTGGTTCTGAAGCGTACCAGGGTGTTCAGCAGAAATGGGACGCT - 180
 41 - A  A  W  G  G  S  G  S  E  A  Y  Q  G  V  Q  Q  K  W  D  A  - 60

181 - ACCGCTACCGAACTGAACAACGCTCTGCAAAACCTGGCTCGTACCATCTCTGAAGCTGGT - 240
 61 - T  A  T  E  L  N  N  A  L  Q  N  L  A  R  T  I  S  E  A  G  - 80

241 - CAGGCTATGGCTTCTACCGAAGGTAACGTAACCGGCATGTTCGCTGGCGGC - 291
 81 - Q  A  M  A  S  T  E  G  N  V  T  G  M  F  A  G  G
```

Ag85B

```
  1 - ATGACCGACGTTTCTCGTAAAATCCGTGCTTGGGGTCGTCGTCTGATGATCGGCACCGCT - 60
  1 - M  T  D  V  S  R  K  I  R  A  W  G  R  R  L  M  I  G  T  A  - 20

 61 - GCTGCTGTTGTTCTGCCGGGTCTGGTTGGTCTGGCTGGTGGTGCTGCTACCGCTGGTGCT - 120
 21 - A  A  V  V  L  P  G  L  V  G  L  A  G  G  A  A  T  A  G  A  - 40

121 - TTCTCTCGTCCGGGTCTGCCGGTTGAATACCTGCAAGTTCCGTCTCCGTCTATGGGTCGT - 180
 41 - F  S  R  P  G  L  P  V  E  Y  L  Q  V  P  S  P  S  M  G  R  - 60

181 - GACATCAAAGTTCAGTTCCAGTCTGGTGGTAACAACTCTCCGGCTGTTTACCTGCTGGAC - 240
 61 - D  I  K  V  Q  F  Q  S  G  G  N  N  S  P  A  V  Y  L  L  D  - 80

241 - GGTCTGCGTGCTCAGGACGACTACAACGGTTGGGACATCAACACCCCGGCTTTTGAATGG - 300
 81 - G  L  R  A  Q  D  D  Y  N  G  W  D  I  N  T  P  A  F  E  W  - 100

301 - TACTACCAGTCTGGTCTGTCTATCGTTATGCCGGTTGGTGGTCAGTCTTCTTTCTACTCT - 360
101 - Y  Y  Q  S  G  L  S  I  V  M  P  V  G  G  Q  S  S  F  Y  S  - 120

361 - GACTGGTACTCTCCGGCTGGTGGTAAAGCTGGTGGTCAGACCTACAAATGGGAAACCTTC - 420
121 - D  W  Y  S  P  A  G  G  K  A  G  G  Q  T  Y  K  W  E  T  F  - 140

421 - CTGACCTCTGAACTGCCGCAGTGGCTGTCTGCTAACCGTGCTGTTAAACCGACCGGCTCT - 480
141 - L  T  S  E  L  P  Q  W  L  S  A  N  R  A  V  K  P  T  G  S  - 160

481 - GCTGCTATCGGTCTGTCTATGGCTGGTTCTTCTGCTATGATCCTGGCTGCTTACCACCCG - 540
```

```
161 - A  A  I  G  L  S  M  A  G  S  S  A  M  I  L  A  A  Y  H  P   - 180

541 - CAGCAGTTCATCTACGCTGGTTCTCTGTCTGCTCTGCTGGACCCGTCTCAGGGTATGGGT - 600
181 - Q  Q  F  I  Y  A  G  S  L  S  A  L  L  D  P  S  Q  G  M  G   - 200

601 - CCGTCTCTGATCGGTCTGGCTATGGGTGACGCTGGTGGTTACAAAGCTGCTGATATGTGG - 660
201 - P  S  L  I  G  L  A  M  G  D  A  G  G  Y  K  A  A  D  M  W   - 220

661 - GGTCCGTCTTCTGACCCGGCTTGGGAACGTAACGACCCGACGCAGCAGATCCCGAAACTG - 720
221 - G  P  S  S  D  P  A  W  E  R  N  D  P  T  Q  Q  I  P  K  L   - 240

721 - GTTGCTAACAACACCCGTCTGTGGGTTTACGGTGGTAACGGCACCCCGAACGAACTGGGT - 780
241 - V  A  N  N  T  R  L  W  V  Y  G  G  N  G  T  P  N  E  L  G   - 260

781 - GGTGCTAACATCCCGGCTGAATTTCTGGAAAACTTCGTTCGTTCTTCTAACCTGAAATTC - 840
261 - G  A  N  I  P  A  E  F  L  E  N  F  V  R  S  S  N  L  K  F   - 280

841 - CAGGACGCTTACAACGCTGCTGGTGGTCACAACGCTGTTTTCAACTTCCCGCCGAACGGC - 900
281 - Q  D  A  Y  N  A  A  G  G  H  N  A  V  F  N  F  P  P  N  G   - 300

901 - ACCCACTCTTGGGAATACTGGGGTGCTCAACTGAACGCTATGAAAGGTGACCTGCAATCT - 960
301 - T  H  S  W  E  Y  W  G  A  Q  L  N  A  M  K  G  D  L  Q  S   - 320

961 - TCTCTGGGTGCTGGTGGCGGC - 981
321 - S  L  G  A  G  G  G
```

Figure S2. Nucleotide sequences of the *de novo* synthesised *pet* gene and heterologous DNA encoding proteins targeted for secretion. A. The nucleotide sequence of the *pet* gene was codon optimised for expression in *E. coli*. Subsequently the sequence was checked for six base pair sites recognised by standard restriction enzymes. The nucleotide sequence was altered to remove existing restriction sites and a limited number of restriction sites were engineered into the gene at defined positions. The precise locations of these restriction sites are indicated by boxes. The amino acid sequence encoded by the synthesised nucleotides is shown. The sequences are coloured according to the domains they represent: the colour scheme adopted in Figure S1 is used. **B.** The nucleotide sequences encoding the portions of the proteins targeted for secretion were codon optimised for expression in *E. coli*. Subsequently, the sequence was edited to remove six base pair sites recognised by standard restriction sites. The amino acid sequence encoded by the synthesised nucleotides is shown.
