# Supplementary material for: A generalised module for the selective extracellular accumulation of recombinant proteins
Source: Microb Cell Fact. 2012 May 28;11:69. doi: 10.1186/1475-2859-11-69 (PMC3419692; doi:10.1186/1475-2859-11-69)
Supplement: Additional file 3 — Figure S3. AT-mediated accumulation of heterologous proteins in the culture medium. [file 1475-2859-11-69-S3.pdf]

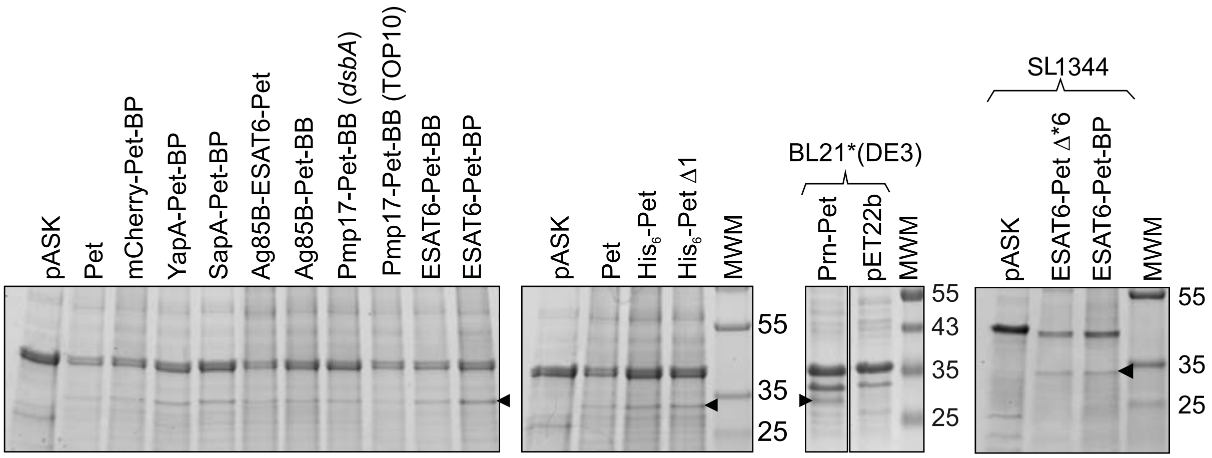

Figure S3. AT-mediated accumulation of heterologous proteins in the culture medium. SDS-PAGE analyses of the OM fractions corresponding to the culture supernatant fractions depicted in Figure 1 are shown. The presence in the OM of the cleaved Pet β-barrel domain is indicated with an arrow. The size of molecular weight markers (MWM, kDa) is indicated on the right of the panel.
