# Supplementary material for: A generalised module for the selective extracellular accumulation of recombinant proteins
Source: Microb Cell Fact. 2012 May 28;11:69. doi: 10.1186/1475-2859-11-69 (PMC3419692; doi:10.1186/1475-2859-11-69)
Supplement: Additional file 4 — Table S1. Mass spectrometry analysis of some recombinant protein fusions with Pet. [file 1475-2859-11-69-S4.pdf]

Table S1. Mass spectrometry analysis of some recombinant protein fusions with Pet.

Fusion protein	Coverage	# PSMs	# Peptides	# AAs	Score	Description
YapA-Pet-BP	35.24	1357	55	1430	5376.35	putative autotransporter protein [Yersinia pestis CA88-4125]
	13.82	638	24	1295	1852.07	RecName: Full=Serine protease pet autotransporter; Contains: RecName: Full=Serine protease pet; AltName: Full=Plasmid-encoded toxin pet; Contains: RecName: Full=Serine protease pet translocator; Flags: Precursor
Ag85B-Pet-BB	41.62	1522	63	1295	5215.18	RecName: Full=Serine protease pet autotransporter; Contains: RecName: Full=Serine protease pet; AltName: Full=Plasmid-encoded toxin pet; Contains: RecName: Full=Serine protease pet translocator; Flags: Precursor
	8.42	72	2	285	255.16	Chain A, Mycobacterium Tuberculosis Antigen 85b With Trehalose
	7.38	72	2	325	255.16	secreted antigen Ag85B [Mycobacterium tuberculosis]
Pertactin-Pet	8.80	572	18	1295	1422.29	RecName: Full=Serine protease pet autotransporter; Contains: RecName: Full=Serine protease pet; AltName: Full=Plasmid-encoded toxin pet; Contains: RecName: Full=Serine protease pet translocator; Flags: Precursor
	22.63	325	12	539	1237.01	Chain A, The Structure Of Bordetella Pertussis Virulence Factor P.69 Pertactin
Pmp17-Pet-BB	39.15	1255	58	1295	4062.94	RecName: Full=Serine protease pet autotransporter; Contains: RecName: Full=Serine protease pet; AltName: Full=Plasmid-encoded toxin pet; Contains: RecName: Full=Serine protease pet translocator; Flags: Precursor
	13.71	237	13	839	664.93	polymorphic outer membrane protein [Chlamydophila abortus S26/3]
ESAT6-Pet-BB	40.93	1471	65	1295	4929.59	RecName: Full=Serine protease pet autotransporter; Contains: RecName: Full=Serine protease pet; AltName: Full=Plasmid-encoded toxin pet; Contains: RecName: Full=Serine protease pet translocator; Flags: Precursor
	38.30	97	2	94	530.53	Chain B, Structure Of The Cfp10-Esat6 Complex From Mycobacterium Tuberculosis
	37.89	59	2	95	310.30	6 kDa early secreted antigenic protein [Mycobacterium ulcerans]
ESAT6-Pet-BP	33.36	1175	46	1295	3500.18	RecName: Full=Serine protease pet autotransporter; Contains: RecName: Full=Serine protease pet; AltName: Full=Plasmid-encoded toxin pet; Contains: RecName: Full=Serine protease pet translocator; Flags: Precursor
	38.30	130	2	94	604.17	Chain B, Structure Of The Cfp10-Esat6 Complex From Mycobacterium Tuberculosis
	37.89	85	2	95	370.05	6 kDa early secreted antigenic protein [Mycobacterium ulcerans]
	37.89	67	2	95	363.46	6 kDa early secretory antigenic target [Mycobacterium kansasii]
	37.89	67	2	95	362.59	Esat6 [Mycobacterium riyadhense]
SapA-Pet-BP	12.66	198	24	1295	542.16	RecName: Full=Serine protease pet autotransporter; Contains: RecName: Full=Serine protease pet; AltName: Full=Plasmid-encoded toxin pet; Contains: RecName: Full=Serine

						protease pet translocator; Flags: Precursor
	4.62	51	3	931	138.88	Flagellar protein [Salmonella enterica subsp. enterica serovar Saintpaul str. SARA23]
mCherry-Pet-BP	10.19	123	17	1295	325.03	RecName: Full=Serine protease pet autotransporter; Contains: RecName: Full=Serine protease pet; AltName: Full=Plasmid-encoded toxin pet; Contains: RecName: Full=Serine protease pet translocator; Flags: Precursor
	47.88	45	16	236	161.22	gb|AAV52164.1| monomeric red fluorescent protein [synthetic construct]
