# Supplementary material for: A generalised module for the selective extracellular accumulation of recombinant proteins
Source: Microb Cell Fact. 2012 May 28;11:69. doi: 10.1186/1475-2859-11-69 (PMC3419692; doi:10.1186/1475-2859-11-69)
Supplement: Additional file 5 — Figure S4. Surface localisation of non cleaved Pet and fusion proteins. [file 1475-2859-11-69-S5.pdf]

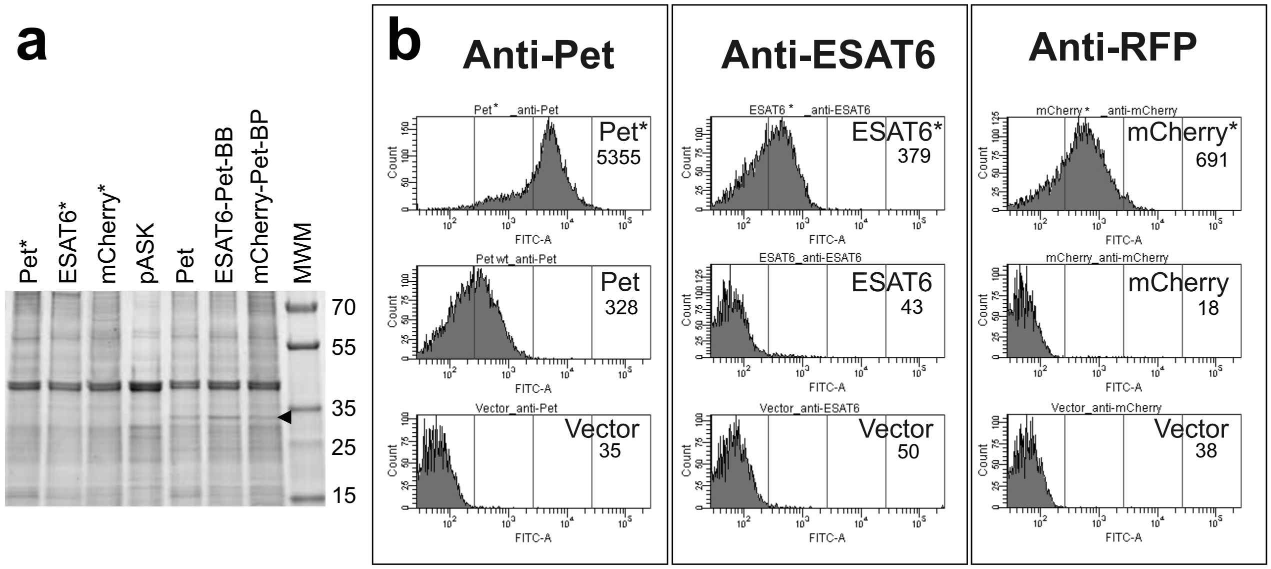

Figure S4. Surface localisation of non cleaved Pet and fusion proteins. A. SDS-PAGE analyses of the OM fractions corresponding to the cultures tested in Figure 2 are shown. OM fractions from non cleaved Pet and fusion proteins (indicated by asterisk, *) do not contain Pet β-barrel doman while those from wt Pet and secreted fusions contain ~30 kDa β-barrel (indicated with an arrow). The size of molecular weight markers (MWM, kDa) is shown on the right of the panel. **B.** Surface localisation of proteins from Figure 2 was assessed by indirect flow cytometry as described in Materials and Methods. *E. coli* TOP10 cultures expressing Pet, ESAT6-Pet-BB and mCherry-Pet-BP and their non cleaved derivatives were labelled with relevant primary antibody as indicated above the FITC histograms. Non cleaved proteins (top panels) show apparent surface localisation compared to the secreted constructs (middle panels) as evident from the increase in green fluorescence intensity. Mean FITC values are shown. Bottom panels show negative controls (empty vector).
