# Supplementary material for: A generalised module for the selective extracellular accumulation of recombinant proteins
Source: Microb Cell Fact. 2012 May 28;11:69. doi: 10.1186/1475-2859-11-69 (PMC3419692; doi:10.1186/1475-2859-11-69)
Supplement: Additional file 6 — Figure S5. Identification of minimal AT module permitting secretion of heterologous proteins to the culture supernatant fraction. [file 1475-2859-11-69-S6.pdf]

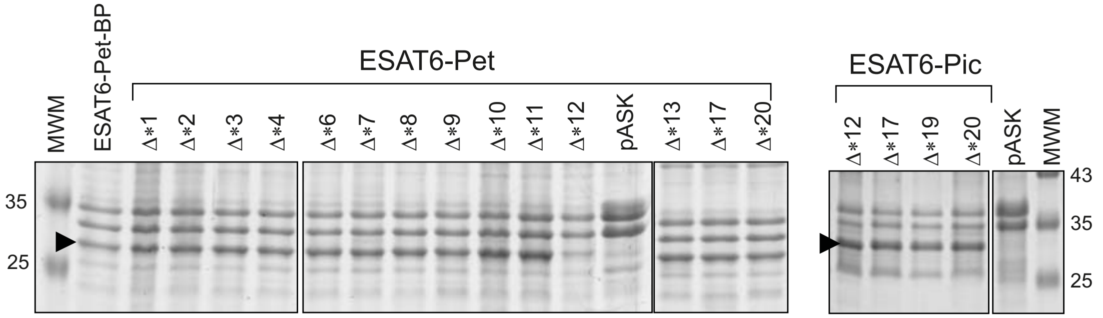

Figure S5. Identification of minimal AT module permitting secretion of heterologous proteins to the culture supernatant fraction. SDS-PAGE analyses of the OM fractions corresponding to some culture supernatant fractions depicted in Figure 3 are shown. The presence in the OM of the cleaved Pet and Pic β-barrel domains is indicated with an arrow. The size of molecular weight markers (MWM, kDa) is indicated.
