# Supplementary material for: A generalised module for the selective extracellular accumulation of recombinant proteins
Source: Microb Cell Fact. 2012 May 28;11:69. doi: 10.1186/1475-2859-11-69 (PMC3419692; doi:10.1186/1475-2859-11-69)
Supplement: Additional file 7 — Figure S6. Impact of conserved amino acids from the AC domain on secretion of heterologous proteins. [file 1475-2859-11-69-S7.pdf]

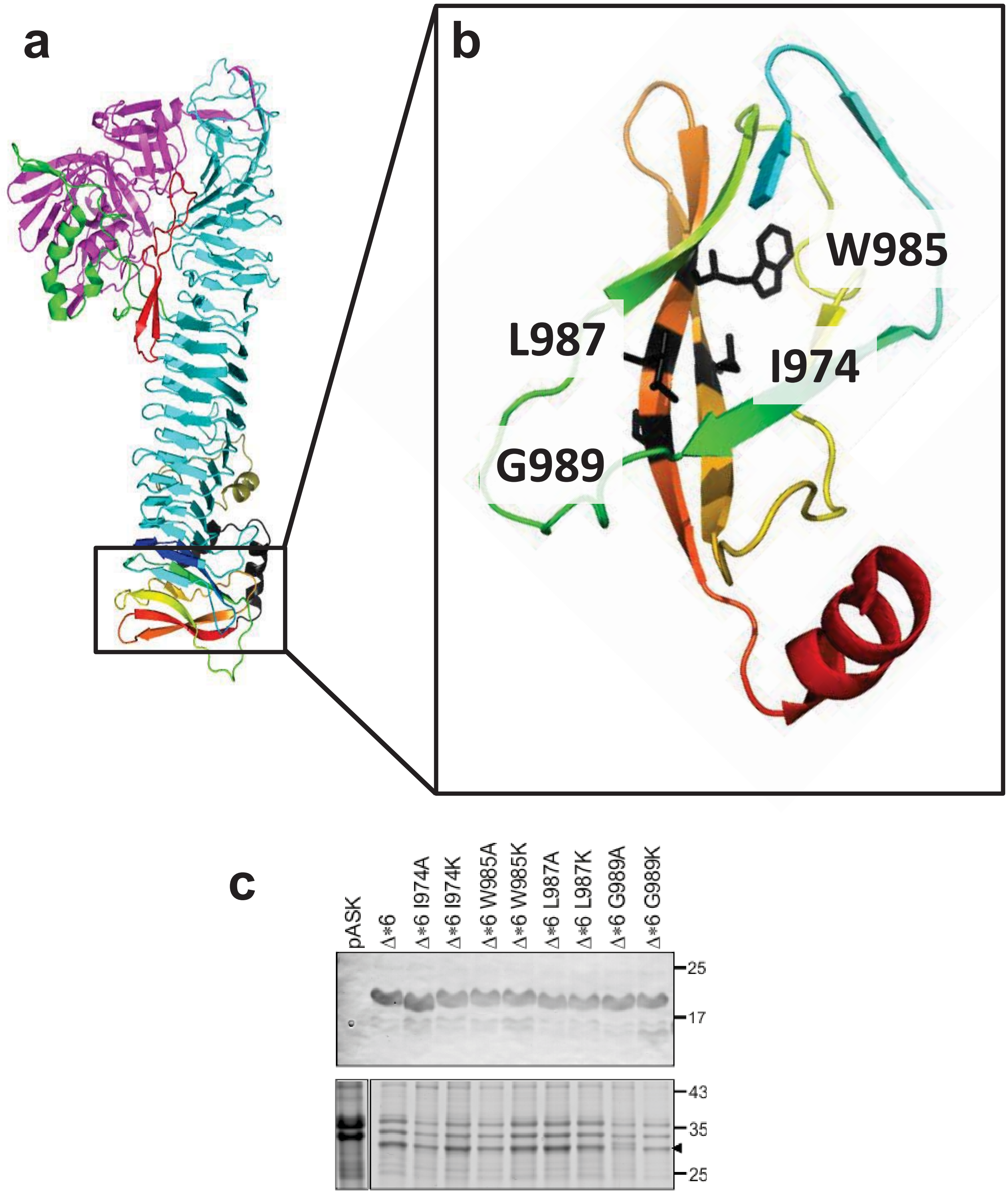

Figure S6. Impact of conserved amino acids from the AC domain on secretion of heterologous proteins. A. Tertiary structure of the AC-α-helix Pet fragment (Pet 920-1018) showing conserved AC domain core residues, I974, W985, L987 and G989. The positions of the conserved residues are highlighted by black sticks. **B.** Analysis of secretion of ESAT-6 fusions with Pet 958-1295 (as in ESAT6-PetΔ*6, Figure 3) carrying mutations in the conserved residues in the AC domain. TCA-precipitated culture supernatants from *E. coli* TOP10 cultures expressing mutant constructs were analysed by Western blotting with anti-ESAT-6. Samples were derived from equal volumes of culture medium. **C.** SDS-PAGE analysis of OM fractions derived from cultures shown in B. The position of the cleaved β-barrel domain is indicated by an arrow.
