# Supplementary material for: A generalised module for the selective extracellular accumulation of recombinant proteins
Source: Microb Cell Fact. 2012 May 28;11:69. doi: 10.1186/1475-2859-11-69 (PMC3419692; doi:10.1186/1475-2859-11-69)
Supplement: Additional file 8 — Figure S7. Comparison of the Pic and Pet SPATE proteins. [file 1475-2859-11-69-S8.pdf]

a

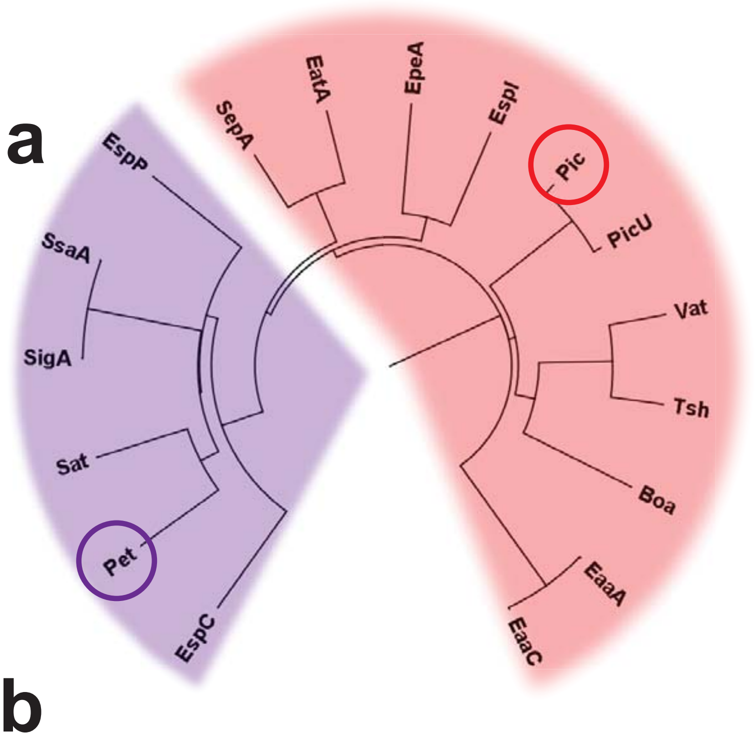

c

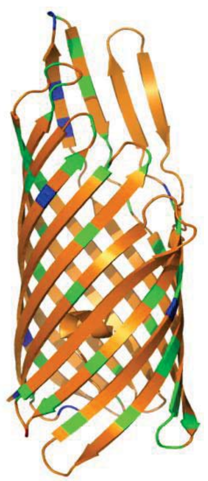

b

```
Pet          TLTVDELTTSNSAFVMRTNTQQADQLIVKNKLEGANNLLLVDFIEKKGNDKNGLNIDLVKAPENTSKDVFKTETQTIGFSDVTPEIKQQEKDGKSVWTLT
Pic          TLTVNKLDATGSDFVLRTDLKNADKINVTEKATGSDNSLNVSFMNNPAQG-QALNIPLVTAPAGTSAEMFKAGTRVTGFSRVTPTLHVDTSGGNTKWILD
Consensus    ****::* ::.* **:**: ::**:: *.:*  *::* * *.*::: .:. :.*** **.** .** ::**: *:. *** *** :: : ..*:: * *

Pet          GYKTVANADAAKKATSLMSGGYKAFLAEVNNLNKRMGDLRDINGEAGAWARIMSGTGSAGGGFSDNYTHVQVGADNKHELDGLDLFTGVTMTYTDSHAGS
Pic          GFKAEADKAAAAKADSFMNAGYKNFMTEVNNLNKRMGDLRDTNGDAGAWARIMSGAGSADGGYSDNYTHVQVGFDKKHELDGVDLFTGVTMTYTDSSADS
Consensus    *:*: *:  ** ** *:*..*** *::************** **:**********:***.**:********** *:******:************* *.*

Pet          DAFSGETKSVGAGLYASAMFESGAYIDLIGKYVHHDNEYTATFAGLGTRDYSSHSWYAGAEVGYRYHVTDSAWIEPQAELVYGAVSGKQFSWKDQGMNLT
Pic          HAFSGKTKSVGGGLYASALFESGAYIDLIGKYIHHDNDYTGNFASLGTKHYNTHSWYAGAETGYRYHLTEDTFIEPQAELVYGAVSGKTFRWKDGDMDLS
Consensus    .****:*****.******:************:****:**..**.***:.*.:********.*****:*:.::************** * *** .*:*:

Pet          MKDKDFNPLIGRTGVDVGKSFSGKDWKVTARAGLGYQFDLFANGETVLRDASGEKRIKGEKDGRMLMNVGLNAEIRDNVRFGLEFEKSAFGKYNVDNAIN
Pic          MKNRDFSPLVGRTGVELGKTFSGKDWSVTARAGTSWQFDLLNNGETVLRDASGEKRIKGEKDSRMLFNVGMNAQIKDNMRFGLEFEKSAFGKYNVDNAVN
Consensus    **::**.**:*****::**:******.******  .:****: *******************.***:***:**:*:**:*****************:*

Pet          ANFRYSF
Pic          ANFRYMF
Consensus    ***** *
```

Figure S7. Comparison of the Pic and Pet SPATE proteins. A. Phylogenetic tree of SPATE family members. Pic clusters with the extracellular proteases (pink shading) and is located on a separate branch to Pet which is grouped with the toxins (purple shading). **B.** Amino acid sequence alignment of Pic and Pet. Identical residues are marked with asterisks and conserved residues with dots. The position of the cleavage site, which separates the passenger domain from the β-domain, is highlighted by red triangles. **C.** Model of Pet β-domain demonstrating the position of amino acid residues which are not identical in Pic. Conserved residues are highlighted by green shading. Non-identical residues are denoted by blue shading.
