# Supplementary material for: A generalised module for the selective extracellular accumulation of recombinant proteins
Source: Microb Cell Fact. 2012 May 28;11:69. doi: 10.1186/1475-2859-11-69 (PMC3419692; doi:10.1186/1475-2859-11-69)
Supplement: Additional file 9 — Table S2. Plasmids used in this study. [file 1475-2859-11-69-S9.pdf]

Table S2. Plasmids used in this study.

Plasmid	Relevant characteristics	Reference
pBADPet	pBADHisA carrying *de novo* synthesized *pet* gene, Amp^r	Leyton et al., 2010
pBADPet*	pBADPet derivative expressing non cleaved Pet mutant, Pet*, with N1018G and D1115G substitutions	Leyton et al., 2010
pASK-IBA33plus	Expression vector, *tet* promoter/operator, Amp^r	IBA BioTAGnology
pET22b	Expression vector, *T7lac* promoter, Amp^r	Novagen
pPic1	pACYC184 plasmids with insertion of *pic* gene from *E. coli* 042	Henderson et al., 1999
pASK-Pet	pASK-IBA33plus expressing Pet, Amp^r	This study
pASK-Pet*	pASK-Pet derivative expressing non cleaved Pet mutant, Pet*, with N1018G and D1115G substitutions	This study
pASK-His_6-Pet	pASK-Pet encoding Pet with a His_6-tag incorporated after signal sequence	This study
pASK-His_6-Pet-ΔD1	Similar to pASK-His_6-Pet; encodes Pet deleted of domain 1	This study
pET-Pet	pET22b expressing Pet, Amp^r	This study
pMA-ESAT6	pMA cloning vector carrying *de novo* synthesized *esxA* gene encoding ESAT-6 from *Mycobacterium tuberculosis*; Amp^r	GenScript/This study
pMA-Ag85B	pMA cloning vector containing *de novo* synthesized *fbpB* gene encoding putative esterase, antigen 85-B (Ag85B) from *Mycobacterium tuberculosis*, Amp^r	GenScript/This study
pET-Pmp17	pET22b containing *de novo* synthesized *pmp17G* gene encoding polymorphic outer membrane protein Pmp17 from *Chlamydophila abortus*, Amp^r	GenScript/This study
pET-SapA	pET22b containing *de novo* synthesized *yaiT* gene encoding putative secreted protein SapA from *Salmonella enterica subsp. enterica serovar Typhimurium*, Amp^r	GenArt/This study
pET-YapA	pET22b containing *de novo* synthesized *yapA* gene encoding putative secreted protein YapA from *Yersinia pestis*, Amp^r	Epoch Life Science /This study
pUC74-mCherry	pUC74 cloning containing *de novo* synthesized *mcherry* gene encoding monomeric red fluorescent protein mCherry, Amp^r	GenScript/This study
pASK-ESAT6-Pet-BB	pASK-Pet with *esxA* insertion between BglII/BstBI sites of *pet*	This study
pASK-ESAT6-Pet*	pASK-Pet-ESAT6-BB expressing non cleaved ESAT6-Pet* fusion with N1018G and D1115G substitutions in Pet translocation domain	This study
pASK-ESAT6-Pet-BP	pASK-Pet with *esxA* insertion between BglII/PstI sites of *pet*	This study
pASK-Ag85B-Pet-BB	pASK-Pet with *fbpB* insertion between *BglII/BstBI* sites of *pet*	This study
pASK-Ag85B-ESAT6-Pet	pASK-Ag85B-Pet-BB with *esxA* insertion between BstBI/PstI sites of *pet*	This study
pASK-Pmp17-Pet-BB	pASK-Pet with insertion of the part of *pmp17G* gene encoding predicted extracellular domain of Pmp17 between *BglII/BstBI* sites of *pet*	This study

pASK-His$_6$-Pmp17-Pet-BB	pASK-Pmp17-Pet-BB with the His$_6$-tag engineered after Pet signal sequence cleavage site	This study
pASK-SapA-Pet-BP	pASK-Pet with insertion of the part of *yaiT* gene encoding predicted extracellular domain of SapA between *BglII/PstI* sites of *pet*	This study
pASK-His$_6$-SapA-Pet-BP	pASK-SapA-Pet-BP with the His$_6$-tag engineered after Pet signal sequence cleavage site	This study
pASK-YapA-Pet-BP	pASK-Pet with insertion of the part of *yapA* gene encoding predicted extracellular domain of YapA between *BglII/PstI* sites of *pet*	This study
pASK-His$_6$-YapA-Pet-BP	pASK-YapA-Pet-BP with the His$_6$-tag engineered after Pet signal sequence cleavage site	This study
pASK-mCherry-Pet-BP	pASK-Pet with *mcherry* insertion between *BglII/PstI* sites of *pet*	This study
pASK-His$_6$-mCherry-Pet-BP	pASK-mCherry-Pet-BP with the His$_6$-tag engineered after Pet signal sequence cleavage site	This study
pASK-mCherry-Pet*	pASK-Pet-mCherry-BP expressing non cleaved mCherry-Pet* fusion with N1018G and D1115G substitutions in Pet translocation domain	This study
pET-Prn-Pet	pET22b containing *de novo* synthesized *prn-pet* sequence encoding extracellular domain of Pertactin (P69.C) from *Bordetella pertussis*	GenScript/This study
pASK-ESAT6-Pet Δ*1	pASK-ESAT6-Pet-BP derivative containing truncated 3' *pet* gene fragment corresponding to Pet 840-1295 protein sequence	This study
pASK-ESAT6-Pet Δ*2	As above, contains *pet* fragment encoding Pet 889-1295	This study
pASK-ESAT6-Pet Δ*3	As above, contains *pet* fragment encoding Pet 925-1295	This study
pASK-ESAT6-Pet Δ*4	As above, contains *pet* fragment encoding Pet 936-1295	This study
pASK-ESAT6-Pet Δ*5	As above, contains *pet* fragment encoding Pet 947-1295	This study
pASK-ESAT6-Pet Δ*6	As above, contains *pet* fragment encoding Pet 958-1295	This study
pASK-ESAT6-Pet Δ*7	As above, contains *pet* fragment encoding Pet 960-1295	This study
pASK-ESAT6-Pet Δ*8	As above, contains *pet* fragment encoding Pet 962-1295	This study
pASK-ESAT6-Pet Δ*9	As above, contains *pet* fragment encoding Pet 964-1295	This study
pASK-ESAT6-Pet Δ*10	As above, contains *pet* fragment encoding Pet 966-1295	This study
pASK-ESAT6-Pet Δ*11	As above, contains *pet* fragment encoding Pet 968-1295	This study
pASK-ESAT6-Pet Δ*12	As above, contains *pet* fragment encoding Pet 971-1295	This study
pASK-ESAT6-Pet Δ*13	As above, contains *pet* fragment encoding Pet 975-1295	This study
pASK-ESAT6-Pet Δ*14	As above, contains *pet* fragment encoding Pet 979-1295	This study
pASK-ESAT6-Pet Δ*15	As above, contains *pet* fragment encoding Pet 982-1295	This study
pASK-ESAT6-Pet Δ*16	As above, contains *pet* fragment encoding Pet 985-1295	This study
pASK-ESAT6-Pet Δ*17	As above, contains *pet* fragment encoding Pet 988-1295	This study
pASK-ESAT6-Pet Δ*18	As above, contains *pet* fragment encoding Pet 994-1295	This study
pASK-ESAT6-Pet Δ*19	As above, contains *pet* fragment encoding Pet 1002-1295	This study
pASK-ESAT6-Pet Δ*20	As above, contains *pet* fragment encoding Pet 1010-1295	This study

pASK-ESAT6-Pic Δ*6	pASK-ESAT6-Pet Δ*6 in which 3' *PstI-HindIII* fragment of *pet* gene was replaced with the equivalent fragment from *pic* gene corresponding to Pic 1035-1372 protein sequence	This study
pASK-ESAT6-Pic Δ*12	pASK-ESAT6-Pet Δ*12 in which 3' *PstI-HindIII* fragment of *pet* gene was replaced with the equivalent fragment from *pic* gene corresponding to Pic 1048-1372 protein sequence	This study
pASK-ESAT6-Pic Δ*17	pASK-ESAT6-Pet Δ*17 in which 3' *PstI-HindIII* fragment of *pet* gene was replaced with the equivalent fragment from *pic* gene corresponding to Pic 1065-1372 protein sequence	This study
pASK-ESAT6-Pic Δ*19	pASK-ESAT6-Pet Δ*19 in which 3' *PstI-HindIII* fragment of *pet* gene was replaced with the equivalent fragment from *pic* gene corresponding to Pic 1079-1372 protein sequence	This study
pASK-ESAT6-Pic Δ*20	pASK-ESAT6-Pet Δ*20 in which 3' *PstI-HindIII* fragment of *pet* gene was replaced with the equivalent fragment from *pic* gene corresponding to Pic 1087-1372 protein sequence	This study
pASK-ESAT6-Pet Δ*6 L987A	pASK-ESAT6-Pet Δ*6 derivative carrying L987→A substitution in AC domain	This study
pASK-ESAT6-Pet Δ*6 L987K	pASK-ESAT6-Pet Δ*6 derivative carrying L987→K substitution in AC domain	This study
pASK-ESAT6-Pet Δ*6 G989A	pASK-ESAT6-Pet Δ*6 derivative carrying G989→A substitution in AC domain	This study
pASK-ESAT6-Pet Δ*6 G989K	pASK-ESAT6-Pet Δ*6 derivative carrying G989→K substitution in AC domain	This study
pASK-ESAT6-Pet Δ*6 W985A	pASK-ESAT6-Pet Δ*6 derivative carrying W985→A substitution in AC domain	This study
pASK-ESAT6-Pet Δ*6 W985K	pASK-ESAT6-Pet Δ*6 derivative carrying W985→K substitution in AC domain	This study
pASK-ESAT6-Pet Δ*6 I974A	pASK-ESAT6-Pet Δ*6 derivative carrying I974→A substitution in AC domain	This study
pASK-ESAT6-Pet Δ*6 I974K	pASK-ESAT6-Pet Δ*6 derivative carrying I974→K substitution in AC domain	This study
