# Supplementary material for: A generalised module for the selective extracellular accumulation of recombinant proteins
Source: Microb Cell Fact. 2012 May 28;11:69. doi: 10.1186/1475-2859-11-69 (PMC3419692; doi:10.1186/1475-2859-11-69)
Supplement: Additional file 10 — Table S3. Primers used in this study. [file 1475-2859-11-69-S10.pdf]

Table S3. Primers used in this study.

Primer	Sequence (5'-3')*
BsaI-pet-F	ATGGTA**GGTCTC**AAATGAACAAAATCTACTCTATC
HindIII-pet-R	GCGC**AAGCTT**TTATCAATGATGATGATGATGATGACC
SacI-pet-F	TTTCT**GAGCTC**GCCAAAAAAGTTATCTGC
PetSS-BglII-AflII-BstBI-R	TTCGAACTTAAGAGATCTAGGTGATGGTGATGGTGATGCGCCGCGTA GATGATGTTGGTGTAAG
BglII-ESAT6-F	GCG**AGATCT**GATGACCGAACAGCAGTGGAAC
BstBI-ESAT6-F	GGCG**TTCGAA**AATGACCGAACAGCAGTGGAAC
BstBI-ESAT6-R	TATA**TTCGAA**TCGCCGCCAGCGAACATG
PstI-ESAT6-R	ATC**CTGCAG**AGCCGCCAGCGAACATGC
BglII-Ag85B-F	CCA**AGATCT**GATGACCGACGTTTCTCGTAA
BstBI-Ag85B-R	TTCC**TTCGAA**TCGCCGCCACCAGCACCCAG
BglII-Pmp17-F	AATA**AGATCT**GAACGACGCGCAGACCGC
BstBI-Pmp17-R	TATA**TTCGAA**TCCGCCAGTTTCGCCGGAG
BglII-SapA-F	GCCG**AGATCT**GACCACCTATGATACCTGGACC
PstI-SapA-R	CCGG**CTGCAG**AGCCATCGCTGTTCATCGCAATG
BglII-YapA-F	ACGT**AGATCT**GGTTTCTCAGATCGCGACCACCG
PstI-YapA-R	TAGC**CTGCAG**ACGCGTTAGACATGTCAACGGTACC
BglII-mCherry-F	GCCG**AGATCT**GATGGTTTCTAAAGGTGAAGAAGAC
PstI-mCherry-R	CCGT**CTGCAG**ATTTATACAGTTCGTCCATACCGC
PstI-TSYQ-del1-F	TATG**CTGCAG**ACACCTCTTACCAGGGTTCTATCAAAGC
PstI-TLTV-del2-F	TATA**CTGCAG**ACACCCTGACCGTTGACGAACTGACC
PstI-NLLL-del3-F	TATA**CTGCAG**ACAACCTGCTGCTGGTCGACTTCATCG
PstI-TPEI-del12-F	TATA**CTGCAG**ACACCCCGGAAATCAAACAGCAGG
PstI-YKAF-del20-F	TATG**CTGCAG**ACTACAAAGCGTTCCTGGCGGAAG
PstI-GNDK-del4-F	TATG**CTGCAG**ACGGTAACGACAAAAACGGTCTGAAC
PstI-VKAP-del5-F	TATA**CTGCAG**ACGTTAAAGCGCCGGAAAACACCTC
PstI-FKTE-del6-F	TATG**CTGCAG**ACTTCAAAACCGAAACCCAGACCATC
PstI-TGYK-del17-F	TATC**CTGCAG**ACACCGGCTACAAAACCGTTGCG
PstI-TETQ-del7-F	TATA**CTGCAG**ACACCGAAACCCAGACCATCGG
PstI-TQTI-del8-F	TATA**CTGCAG**ACACCCAGACCATCGGTTTCTCTG
PstI-TIGF-del9-F	CATA**CTGCAG**ACACCATCGGTTTCTCTGACGTTACC
PstI-GFSD-del10-F	TGTA**CTGCAG**ACGGTTTCTCTGACGTTACCCCG
PstI-SDVT-del11-F	TCTG**CTGCAG**ACTCTGACGTTACCCCGGAAATC
PstI-KQQE-del13-F	GGCA**CTGCAG**ACAAACAGCAGGAAAAAGACGGTAAATC
PstI-KDG-del14-F	GGCA**CTGCAG**ACAAAGACGGTAAATCTGTTTGGACC
PstI-KSV-del15-F	GGCA**CTGCAG**ACAAATCTGTTTGGACCCTGACC
PstI-WTL-del16-F	TATA**CTGCAG**ACTGGACCCTGACCGGCTACAAAACC
PstI-ANAD-del18-F	GGCA**CTGCAG**ACGCGAACGCGGACGCGGCG
PstI-ATSL-del19-F	GGCA**CTGCAG**ACGCGACCTCTCTGATGTCTGGTGG
SbfI-FKAG-Pic-del6-F	GGCA**CCTGCAGG**CTTTAAGGCCGGCACCCGGGTGAC
SbfI-TPTL-Pic-del12-F	GGCA**CCTGCAGG**CACCCCAACCCTGCATGTTGATACC
SbfI-DGFK-Pic-del17-F	GGCA**CCTGCAGG**CGATGGTTTTAAAGCGGAGGCTGATAAAG
SbfI-ADSF-Pic-del19-F	GGCA**CCTGCAGG**CGCTGACAGTTTCATGAATGCCGGG
SbfI-YKNF-Pic-del20-F	GGCA**CCTGCAGG**CTATAAAAACTTCATGACGGAAGTTAAC
HindIII-Pic-end-R	GCGC**AAGCTT**TCAGAACATATACCGGAAATTCGCG
pASK-IBA33+ For seq	GAGTTATTTACCACTCCCT
pASK-IBA33+ Rev seq	CGCAGTAGCGGTAAACG
pet600For seq	ATCTTCTGCGAACTACC
pet1320For seq	GGTGGTTTCATCTTCGA
pet2880For seq	TCTTCAACACCCTGACC
pet3660For seq	TTCTCACTCTTGGTACG
pet2041For seq (Spe/BsrG)	TTCTGACCTGTCTCAGC
pet2710 For seq	TACCAGGGTTCTATCAAAGCG
pet2731 Rev seq	CCGCTTTGATAGAACCCTGG

pet3401 Rev seq	TCCAGACCGTCCAGTTCG
**Site directed mutagenesis primers**	
AC_Ile to Ala_F	GACGTTACCCCGGAAGCGAAACAGCAGGAAAAAG
AC_Ile to Ala_R	CTTTTTCCTGCTGTTTCGCTTCCGGGGTAACGTC
AC_Ile to Lys_F	GACGTTACCCCGGAAAAAAAACAGCAGGAAAAAG
AC_Ile to Lys_R	CTTTTTCCTGCTGTTTTTTTTCCGGGGTAACGTC
AC_W to Ala_F	AAAGACGGTAAATCTGTTGCGACCCTGACCGGCTAC
AC_W to Ala_R	GTAGCCGGTCAGGGTCGCAACAGATTTACCGTCTTT
AC_W to Lys_F	AAAGACGGTAAATCTGTTAAAACCCTGACCGGCTAC
AC_W to Lys_R	GTAGCCGGTCAGGGTTTTAACAGATTTACCGTCTTT
AC_Leu to Ala_F	GTAAATCTGTTTGGACCGCGACCGGCTACAAAACC
AC_Leu to Ala_R	GGTTTTGTAGCCGGTCGCGGTCCAAACAGATTTAC
AC_Leu to Lys_F	GTAAATCTGTTTGGACCAAAACCGGCTACAAAACC
AC_Leu to Lys_R	GGTTTTGTAGCCGGTTTTGGTCCAAACAGATTTAC
AC_Gly to Ala_F	CTGTTTGGACCCTGACCGCGTACAAAACCGTTGCG
AC_Gly to Ala_R	CGCAACGGTTTTGTACGCGGTCAGGGTCCAAACAG
AC_Gly to Lys_F	CTGTTTGGACCCTGACCAAATACAAAACCGTTGCG
AC_Gly to Lys_R	CGCAACGGTTTTGTATTTGGTCAGGGTCCAAACAG

* Restriction sites engineered on primers are in **bold font**; mutated nucleotides are in grey
